# Supplementary material for: SPT6L, a newly discovered ancestral component of the plant RNA-directed DNA methylation pathway
Source: Front Plant Sci. 2024 Mar 21;15:1372880. doi: 10.3389/fpls.2024.1372880 (PMC10991848; doi:10.3389/fpls.2024.1372880)

Supplemental materials

Supplemental Figures

Supplemental Fig. S1: Analysis of SPT6L loci indicates that SPT6L is a part of the Pol V complex

Supplemental Fig. S2: Characterization of Pol V loci occupied by SPT6L

Supplemental Fig. S3: Mode of DNA methylation, types of sRNAs and genomic elements at Pol V loci occupied by SPT6L

Supplemental Fig S4: Evolution of SPT6L, other relevant AGO-hook proteins and DNA methyltransferases in *Viridiplantae*

Supplemental Tables

Supplemental Table S1: Datasets used in this study

Supplemental Table S2: AlphaFold-Multimer (AFM) prediction scores (ipTM) for complex formation between SPT6L and relevant Pol II subunits and SPT6L and all NRPB7 paralogues. Scores for all five models generated by AFM are shown. List of used UniProt chain IDs. It should be noted that the two RdDM-related paralogues of NRPB7 are annotated in the TAIR10/Araport11 as NRPD7 and NRPE7, but NRPE7 is present in both Pol IV and Pol V complexes (Law et al. 2011) and more importantly their diversification is specific for the *Brassicaceae* family, other species have only one NRPD/E7 protein (Wang and Ma, 2015).

Supplemental Table S3: Table with underlying data for Fig. 3A,B and Supplemental Fig. S3A,B.

Supplemental Table S4: Table with underlying data for Fig. 3C and Supplemental Fig. S3C.

Supplemental Fig. S1: Analysis of SPT6L loci indicates that SPT6L is a part of the Pol V complex

Supplemental figures for Fig. 1. (A) Three representative genomic regions with SPT6L ChIP-seq and sRNA-seq data with indicated loci which are significantly enriched for these features and categorisation of these loci based on their overlaps. Pol V ChIP-seq data are shown to illustrate its coverage at the SPT6L and sRNA loci. (B) Boxplots with z-score of ChIP-seq coverage for various chromatin marks at loci presented in Fig. 1A. (C) Metaplots showing the ChIP-seq data of selected chromatin marks, WGBS data and ATAC-seq data (all in WT) for loci presented in Fig. 1A (± 1 kbp of adjacent regions). Randomized SPT6L∩sRNA loci are used as background. This is extended characterization of data from Fig. 1B and Supplemental Fig. S1B. (D) Clustered heatmap for SPT6L∩sRNA loci of data presented in Fig. 1B and Supplemental Fig. S1B (z-score is plotted in the graph). (E) Metaplots showing the ChIP-seq data of several RdDM proteins and Pol II for loci presented in Fig. 1A (± 1 kbp of adjacent regions). Randomized SPT6L∩sRNA loci are used as background. This is extended characterization of data from Fig. 1C. (E) Clustered heatmap for SPT6L∩sRNA loci of data presented in Fig. 1C (z-score is plotted in the graph). (G) SPT6L is present at Pol V loci, but not Pol IV loci. Metaplot showing ChIP-seq data for SPT6L at Pol IV loci with overlap with Pol V loci (Pol IV∩Pol V) or without it (Pol IV(-)Pol V) and Pol V loci without overlap with Pol IV loci (Pol V(-)Pol IV). Randomized Pol IV∩Pol V loci are used as background. (H) Venn diagram showing overlaps between Pol IV, Pol V and SPT6L loci, these overlaps are presented in the panel G. (I) Overlay of the AlphaFold-Multimer predictions for the SPT6LxNRPB7 (light blue/green) and SPT6LxNRPE7 (dark blue/orange) complexes. Note that the SH2 domain of SPT6L is connected with a flexible linker to the rest of the SPT6L and therefore its position is not fixed in the structure, as can be seen also from Fig. 1F. The unstructured parts of SPT6L are not shown (300 aa residues from N-terminus and 210 aa residues from C-terminus).

Fig. S1A

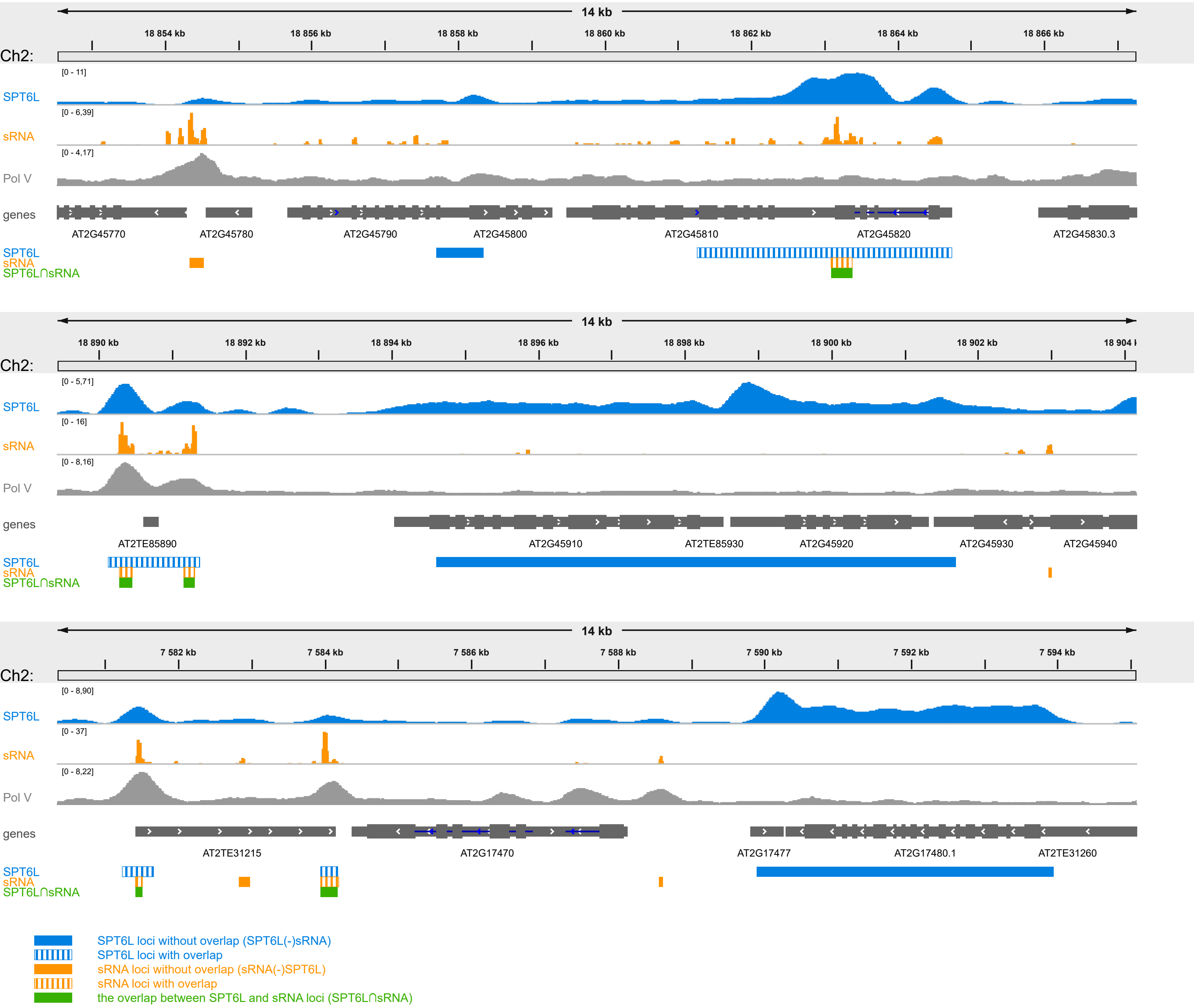

Fig. S1B

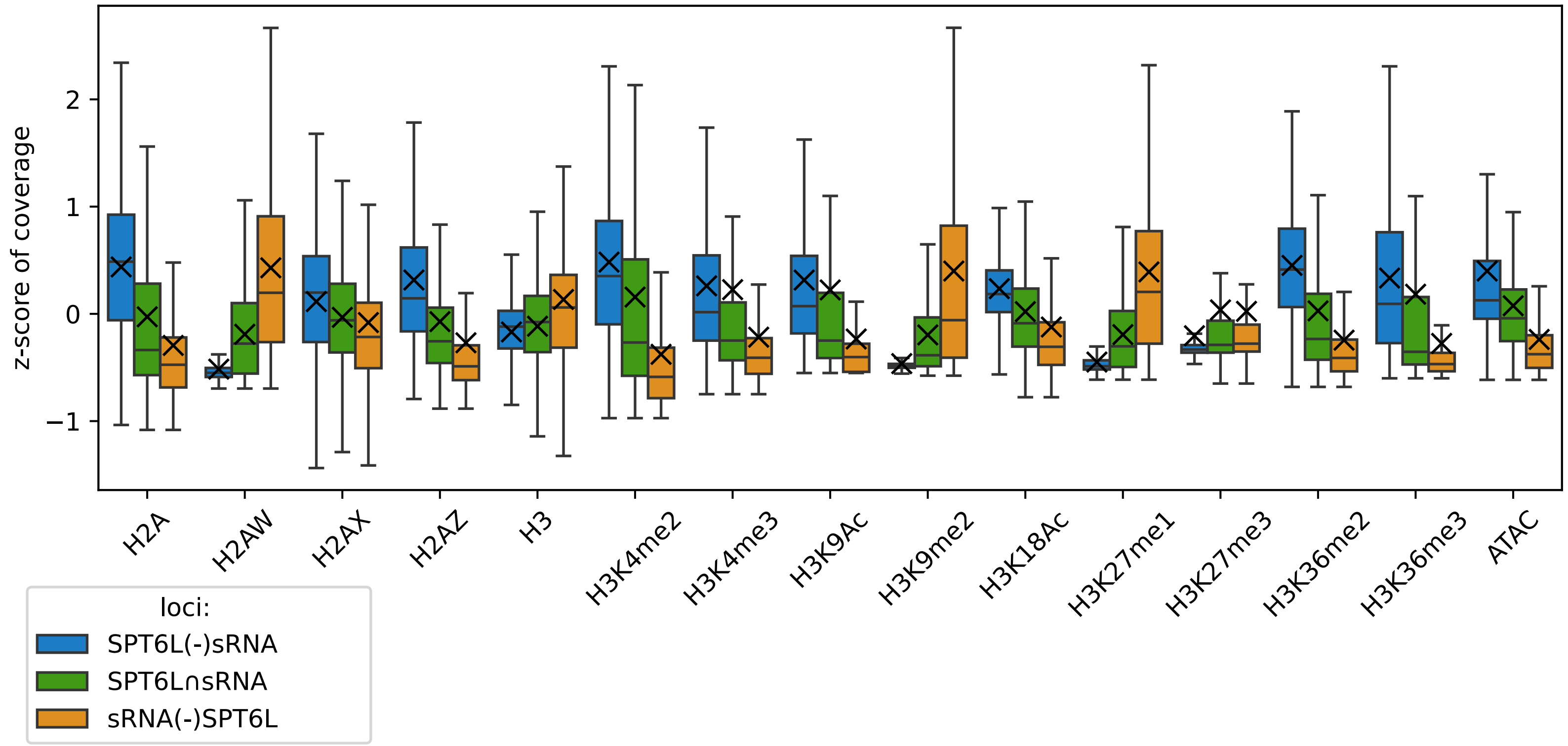

Fig. S1C

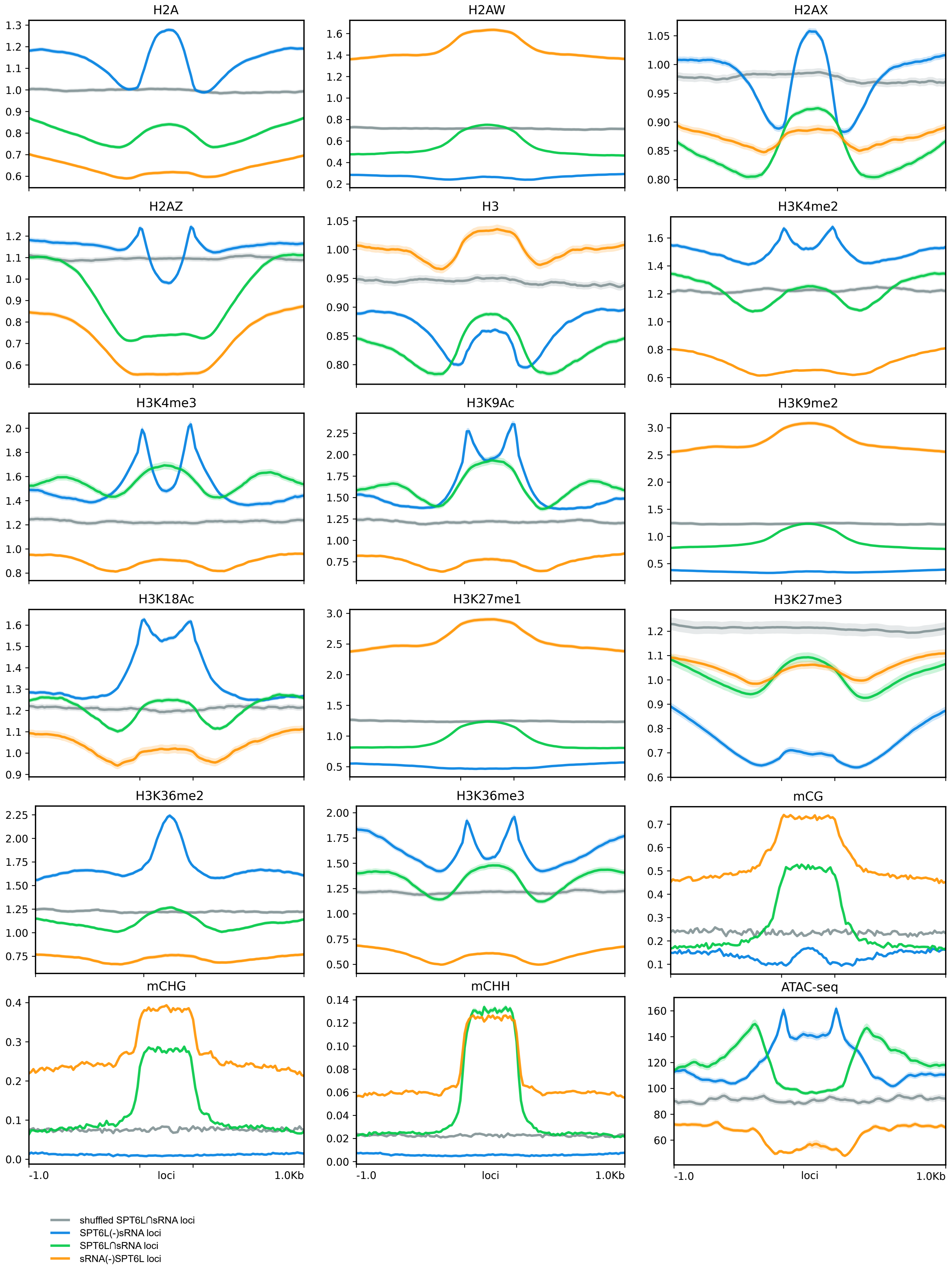

H2A
H2AW
H2AX
H2AZ
H3
H3K4me2
H3K4me3
H3K9Ac
H3K9me2
H3K18Ac
H3K27me1
H3K27me3
H3K36me2
H3K36me3
mCG
mCHG
mCHH
ATAC-seq
-1.0
loci
1.0Kb
shuffled SPT6L∩sRNA loci
SPT6L(-)sRNA loci
SPT6L∩sRNA loci
sRNA(-)SPT6L loci

Fig. S1D

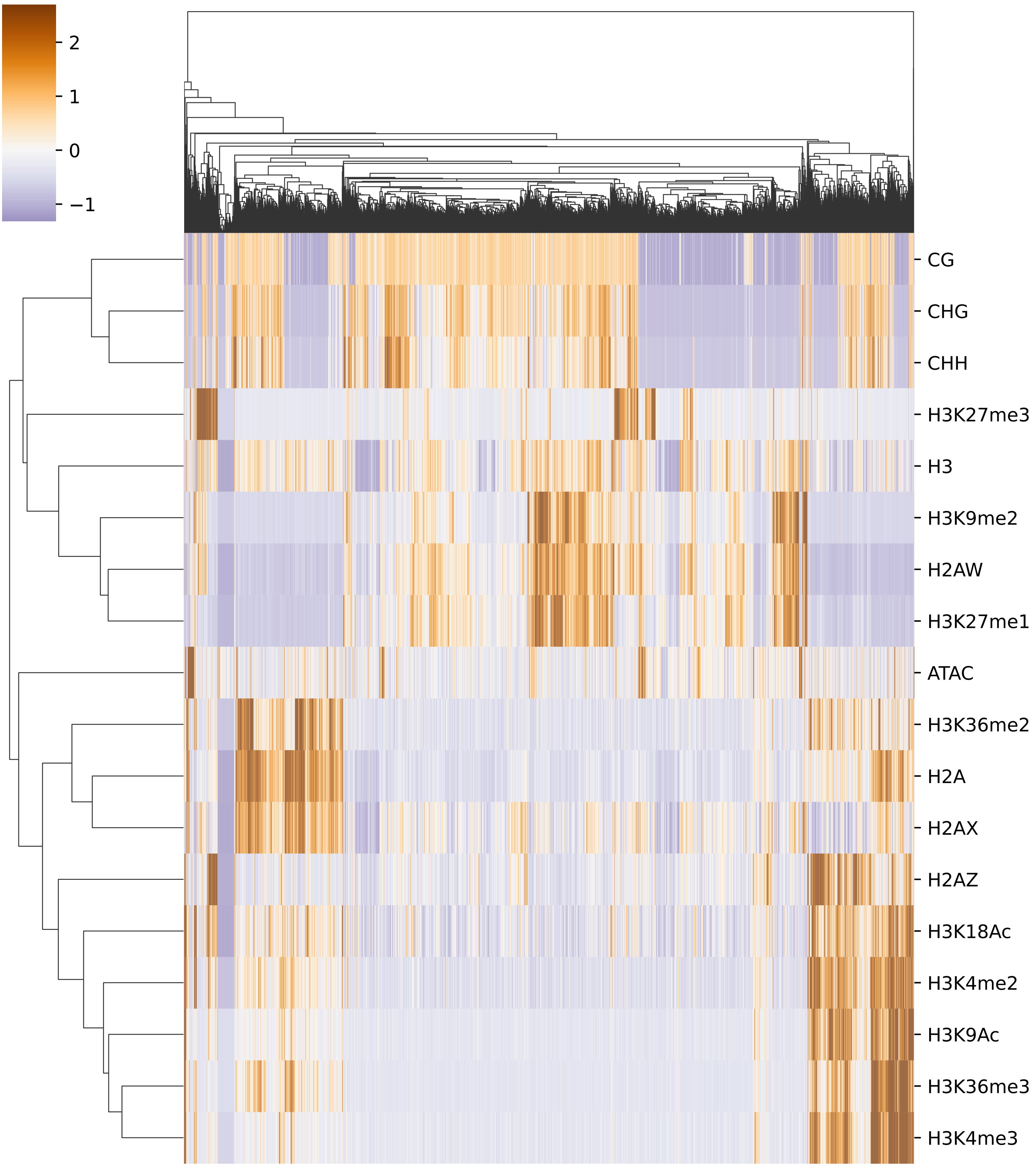
2
1
0
−1
CG
CHG
CHH
H3K27me3
H3
H3K9me2
H2AW
H3K27me1
ATAC
H3K36me2
H2A
H2AX
H2AZ
H3K18Ac
H3K4me2
H3K9Ac
H3K36me3
H3K4me3

Fig. S1E

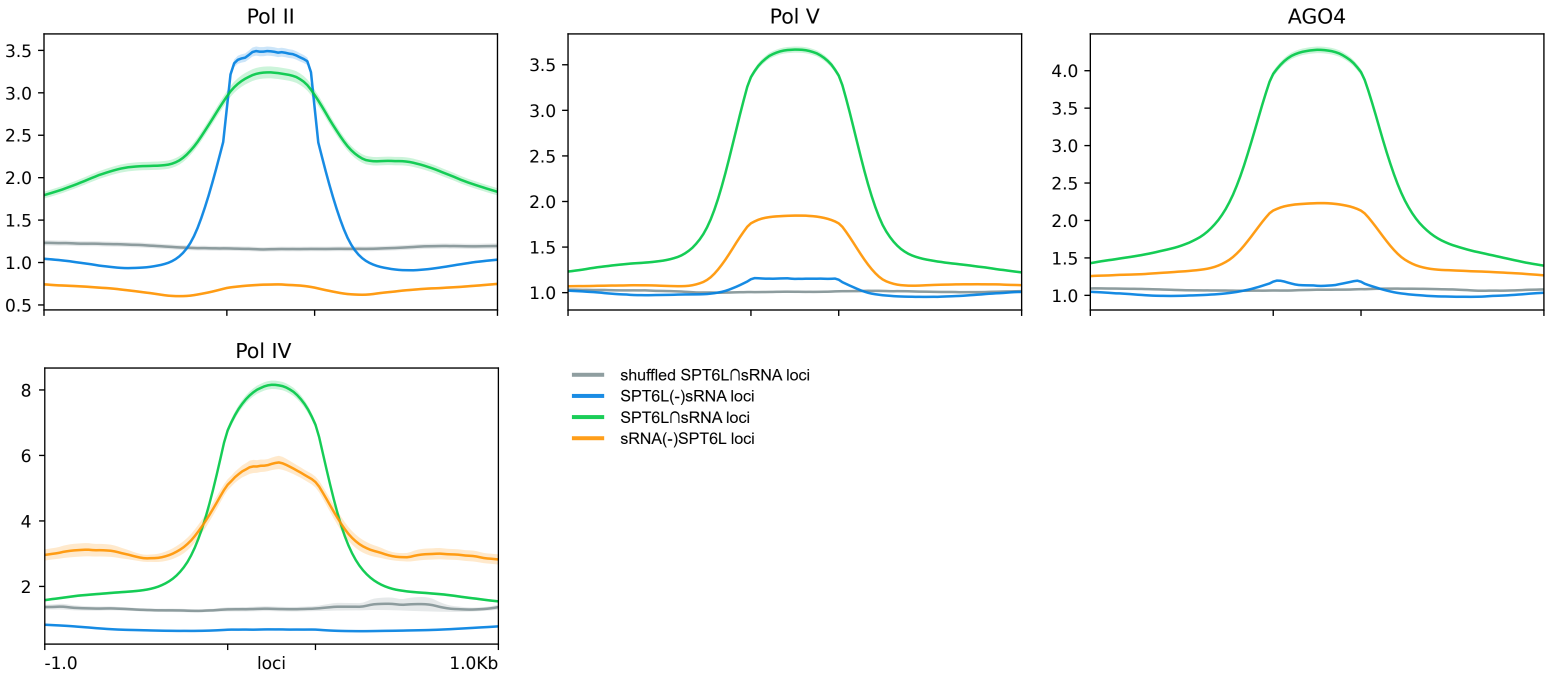

Fig. S1F

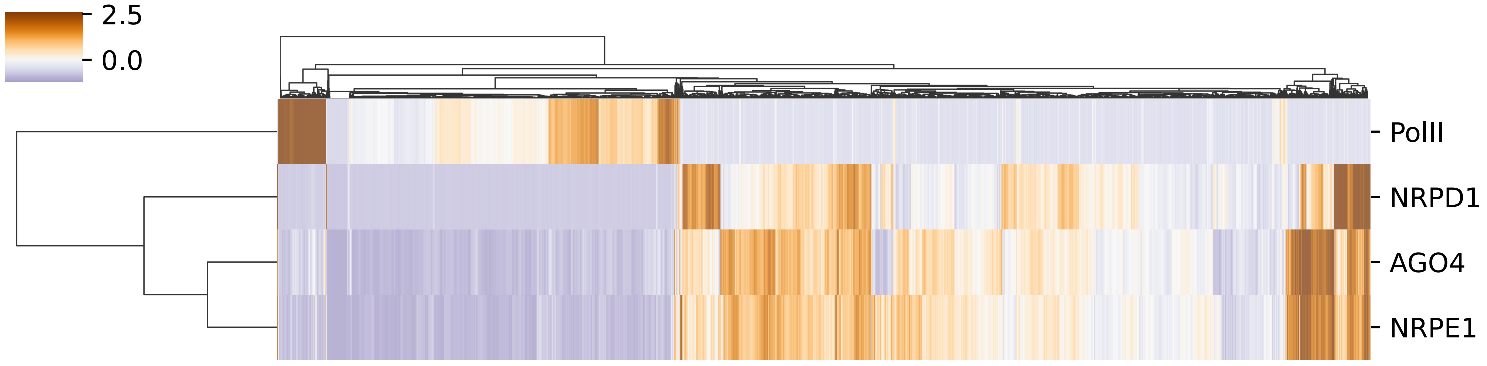

Fig. S1G

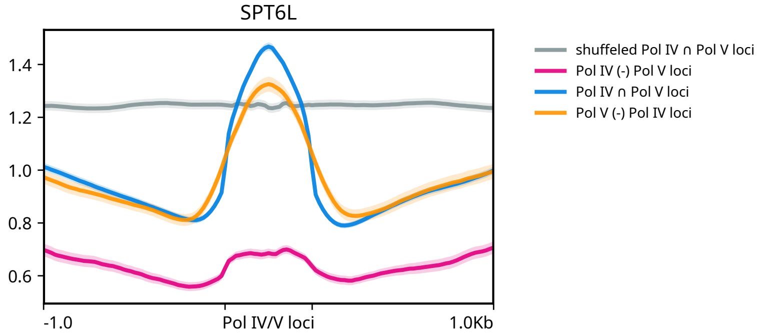

Fig. S1H

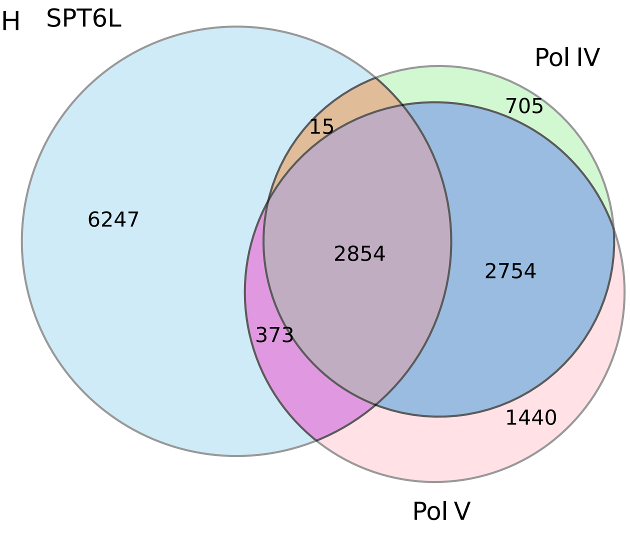

Fig. S1I

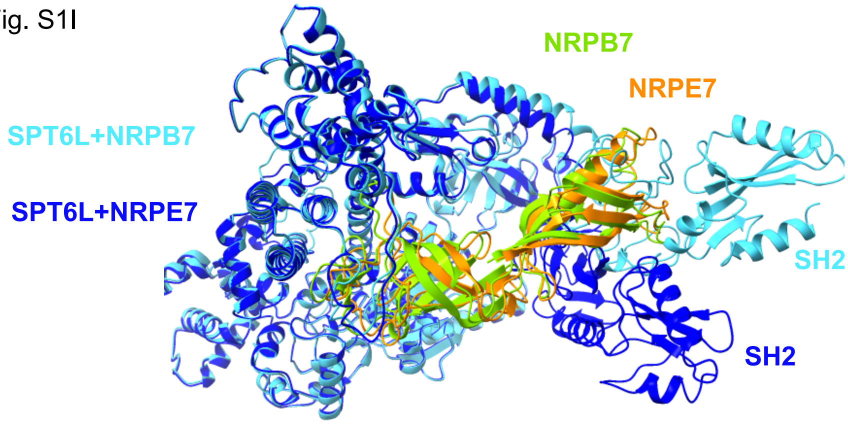

Supplemental Fig. S2: Characterization of Pol V loci occupied by SPT6L

Supplemental figures for Fig. 2. (A) Distribution of Pol II, SPT6L, Pol V and subcategories of Pol V loci along chromosomes of *A. thaliana*. (B) Three representative genomic regions with Pol II (NRPB1), SPT6L and Pol V (NRPE1) ChIP-seq data with indicated loci which are significantly enriched for these proteins. Pol V loci are categorized according to Fig. 2A. (C,D) Extended version of Fig. 2 to show how the analyzed loci are affected by Pol II. (C) Boxplots showing the lengths and GC content of the four subcategories of Pol V loci. Note generally lower GC content at Pol V loci than at Pol II loci that are shown for comparison. (D) Enrichment/depletion of chromatin marks and RdDM components at P5(+)SPT6L loci calculated as log2(P5(+)SPT6L/P5(-)SPT6L) and P5(+)SPT6L(-)P2 loci calculated as log2(P5(+)SPT6L(-)P2/P5(-)SPT6L(-)P2). (E,F) Metaplots showing ChIP-seq data (underlying data for Fig. 2C,E) for (E) various chromatin marks and (F) RdDM components at the four subcategories of Pol V loci. Metaplots contain ± 1 kbp of adjacent regions, randomized P5 loci are used for background. Significance is indicated by asterisks: $p < 0.05$ = *; $p < 0.005$ = **; $p < 0.0005$ = ***; NS = not significant; in Supplemental Fig. S2D the statistics were calculated from the normalized coverages at the P5(+)SPT6L and P5(-)SPT6L or P5(+)SPT6L(-)P2 and P5(-)SPT6L(-)P2 loci from which the log2 ratio was calculated.

Fig. S2A

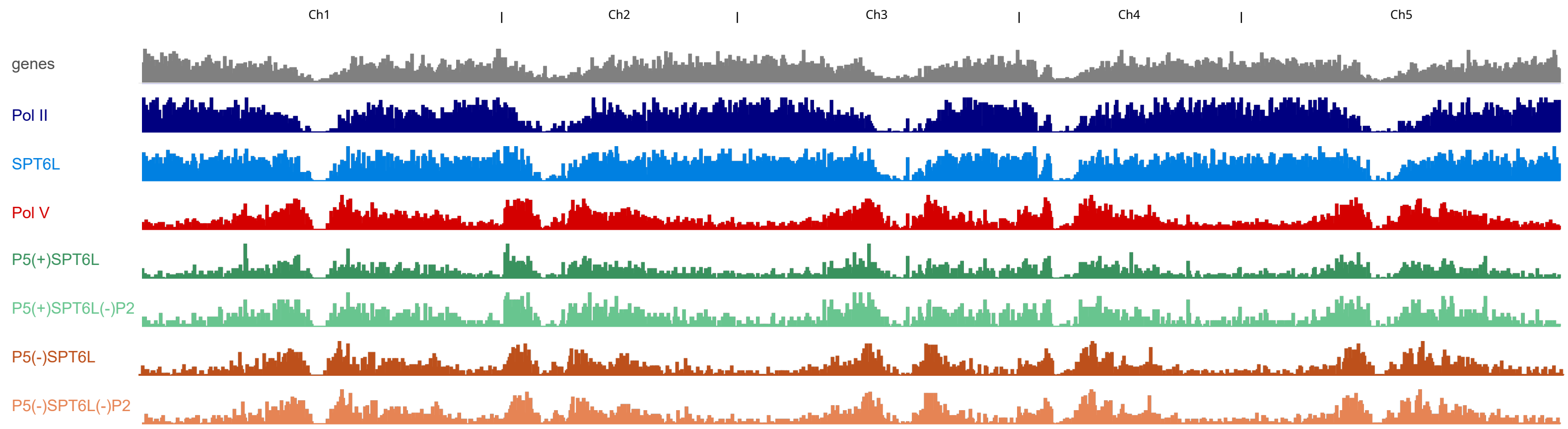

Fig. S2B

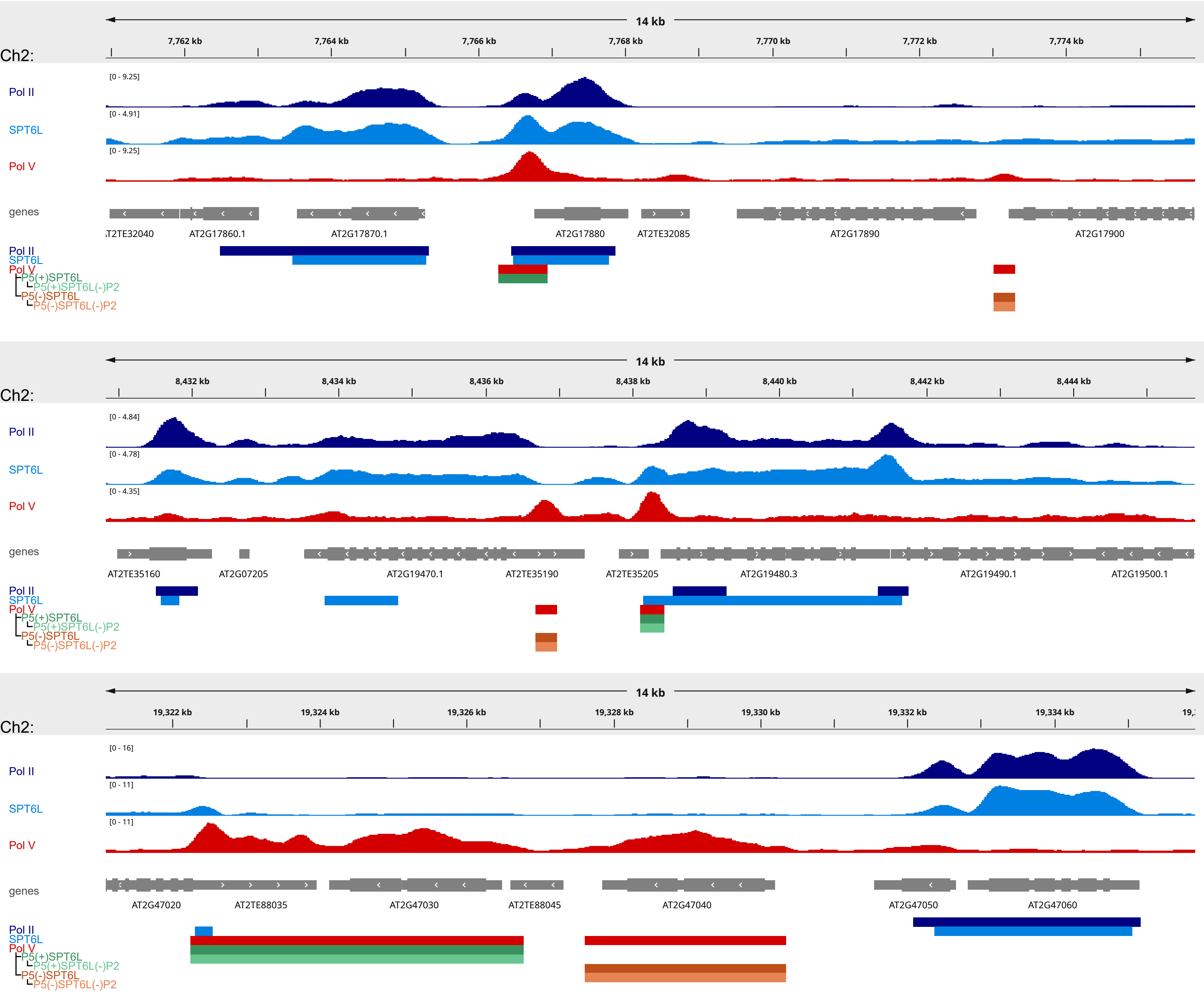

Fig. S2C

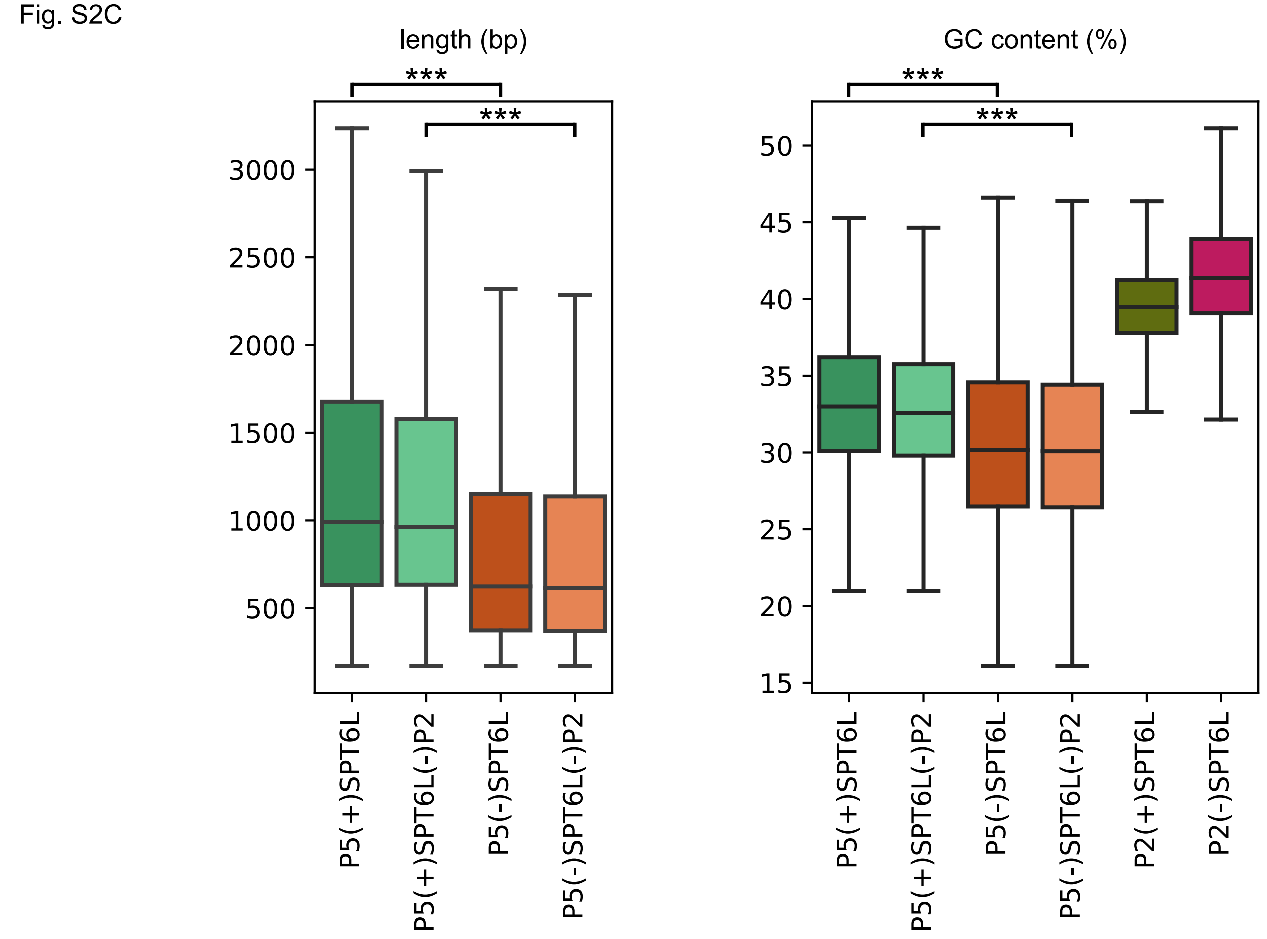

Fig. S2D

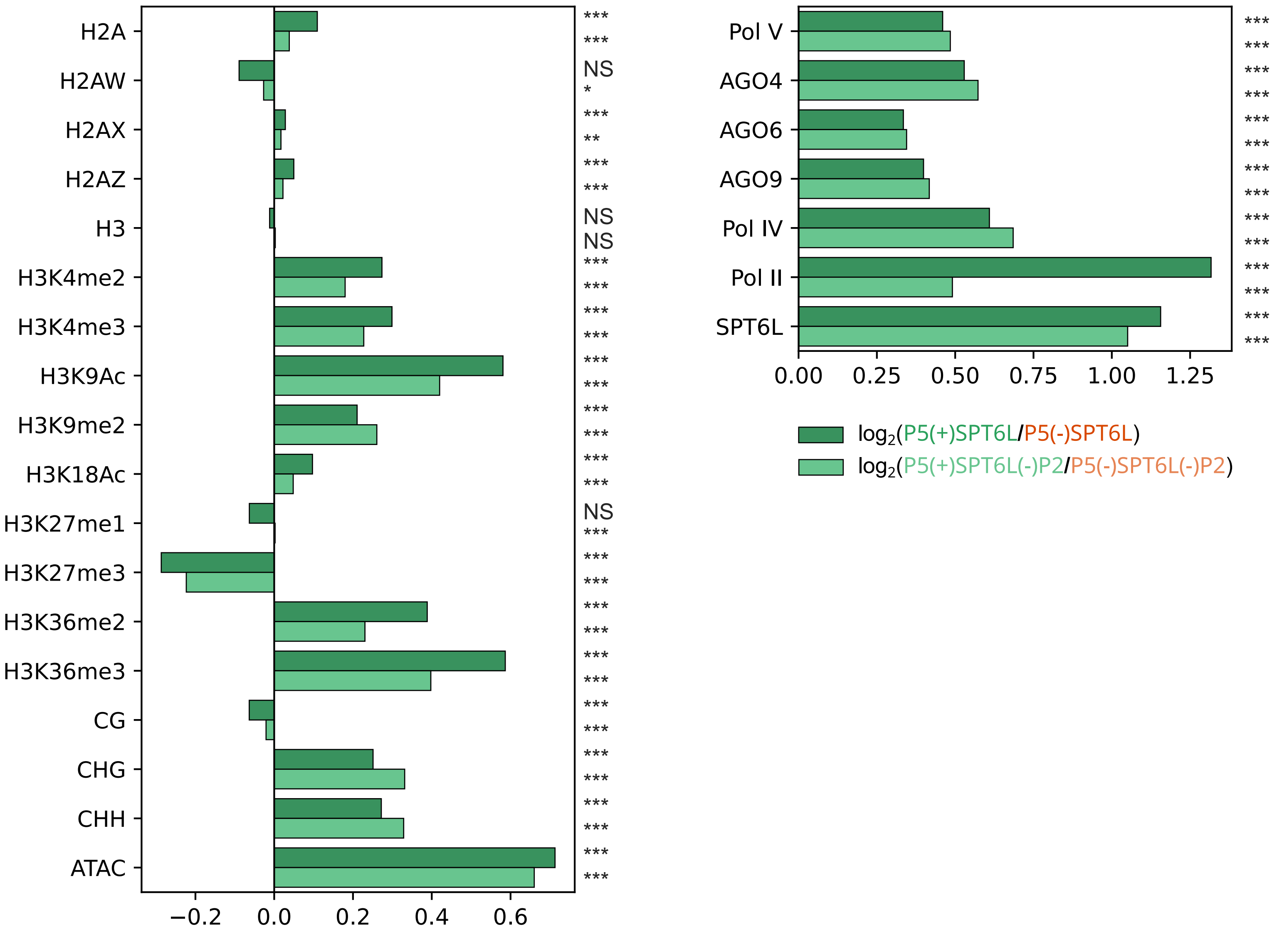

Fig. S2E

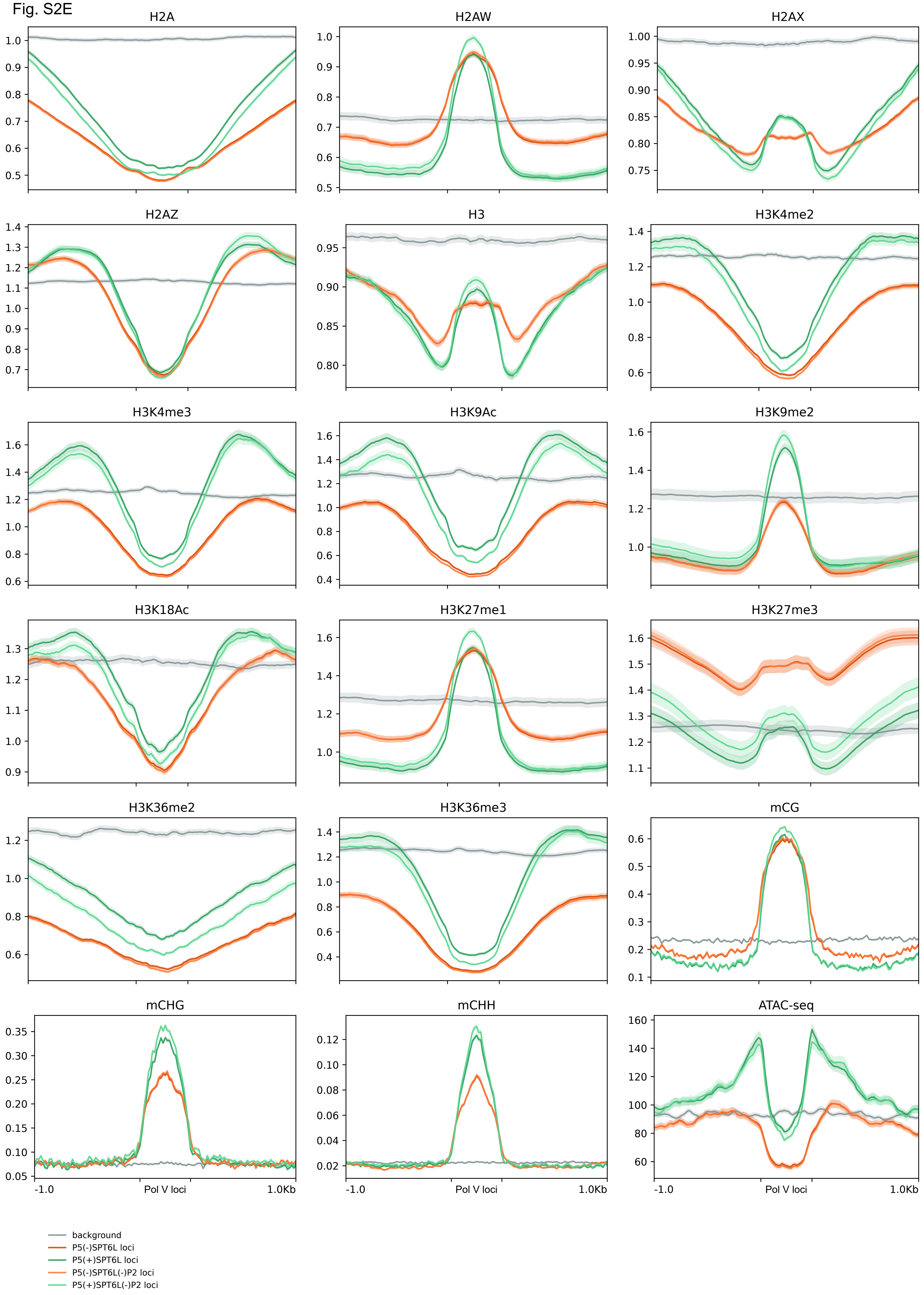
H2A
H2AW
H2AX
H2AZ
H3
H3K4me2
H3K4me3
H3K9Ac
H3K9me2
H3K18Ac
H3K27me1
H3K27me3
H3K36me2
H3K36me3
mCG
mCHG
mCHH
ATAC-seq
-1.0
Pol V loci
1.0Kb
background
P5(-)SPT6L loci
P5(+)SPT6L loci
P5(-)SPT6L(-)P2 loci
P5(+)SPT6L(-)P2 loci

Fig. S2F

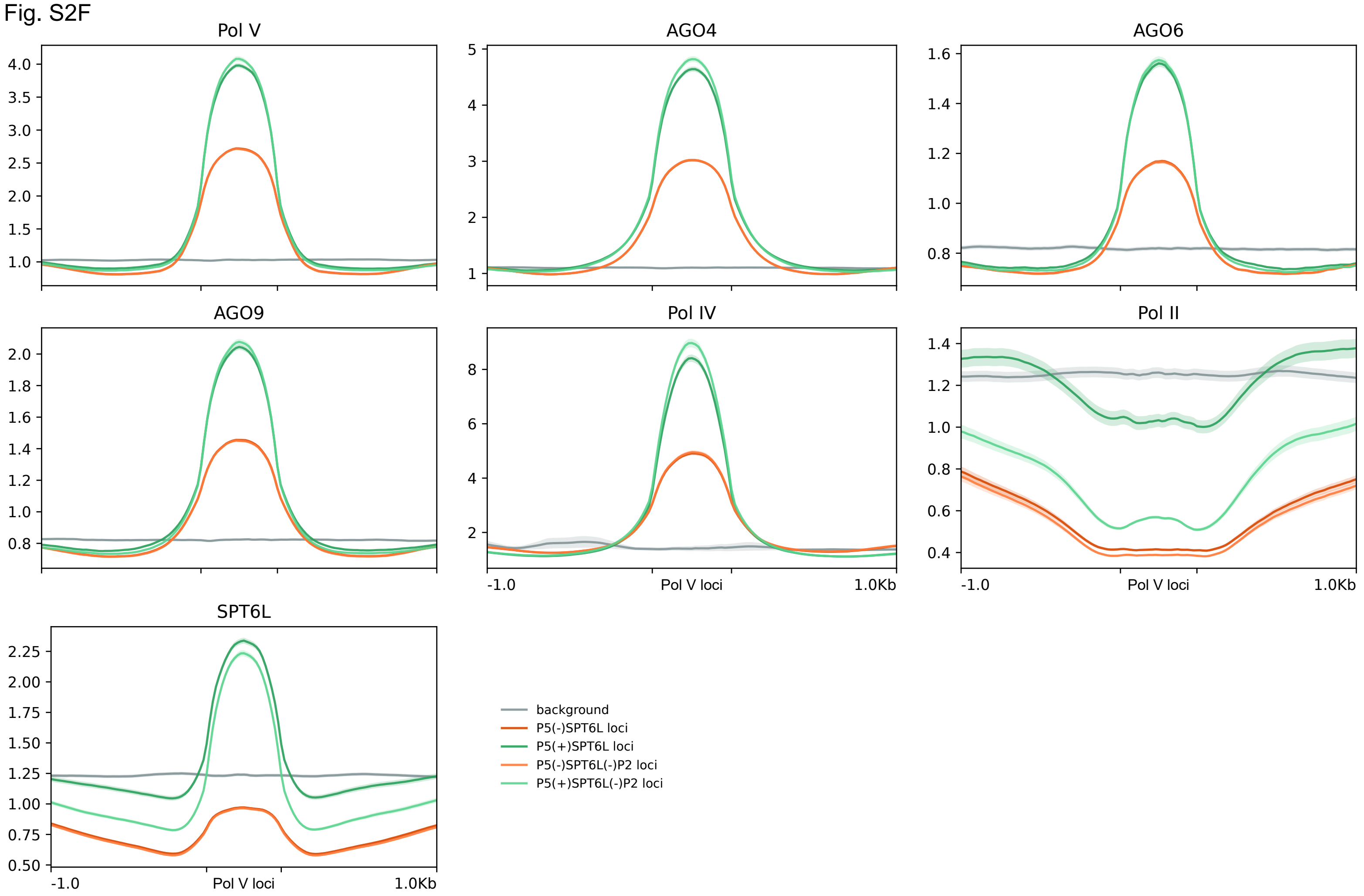

Pol V
AGO4
AGO6
AGO9
Pol IV
Pol II
SPT6L
-1.0
Pol V loci
1.0Kb
background
P5(-)SPT6L loci
P5(+)SPT6L loci
P5(-)SPT6L(-)P2 loci
P5(+)SPT6L(-)P2 loci

Supplemental Fig. S3: Mode of DNA methylation, types of sRNAs and genomic elements at Pol V loci occupied by SPT6L

Extended version of Fig. 3 to show how the analyzed loci are affected by Pol II. (A,B) Enrichment/depletion of differentially methylated regions (DMRs) at P5(+)SPT6L(-)P2 loci in datasets/samples from Zhang et al. 2018 containing whole genome methylation data for various mutants and some tissues. Violin plots show how the individual samples are enriched at P5(+)SPT6L(-)P2 loci (i.e. whether DMRs from the sample tend to overlap the P5(+)SPT6L(-)P2 loci more than the P5(-)SPT6L(-)P2 loci): (A) sample assessment based on DMR type (cytosine context and hyper-/hypomethylation) and by (B) the sample category that is shown only for hypo-CHH DMRs in the sample. Only datasets significantly enriched/depleted for the P5(+)SPT6(-)P2 loci are shown. In panel A the width of the violin plot is scaled to the number of samples and vertical lines indicate median and quartiles, in panel B the plots are not scaled and vertical lines indicate individual datasets; position of *nrpe1* mutant datasets is outlined below by the red rectangle. Data for individual datasets and their classification can be found in Supplemental Table S3. (C) Heatmap with enrichment of sRNA at P5(+)SPT6L(-)P2 loci for indicated samples and sRNA lengths calculated as log2(P5(+)SPT6L(-)P2/P5(-)SPT6L(-)P2). Analyzed sRNA-seq datasets originate either from AGO immunoprecipitation or from total sRNA samples in case of "input" and WT, and *nrpe1* and *nrpd1* mutants. Samples outlined within the same rectangle come from the same experiment (these are well comparable with each other). Individual replicates are shown in Supplemental Table S4. (D) Enrichment/depletion of overlap of P5(+)SPT6L and P5(+)SPT6L(-)P2 loci at different types of genomic elements (see Methods for genomic elements definition) and TE categories as defined by Panda et al. 2016: TE family, TE expression, Type of TE silencing in WT (either canonical PolIV-RdDM or various non-canonical RdDM pathways, non-RdDM pathways or not determined, ND), Type of TE silencing in *ddm1*. The "n" indicates the total number of genomic elements or TEs in a given category. (E) Characteristic features of TEs overlapping P5(+)SPT6L, P5(-)SPT6L, P5(+)SPT6L(-)P2 and P5(-)SPT6L(-)P2 loci, features adapted from Panda et al. 2016: TE copy number, TE length, TE gene distance and TE centromere distance. Significance is indicated by asterisks: $p < 0.05$ = *; $p < 0.005$ = **; $p < 0.0005$ = ***; NS = not significant. The significance indicates either enrichment of P5(+)SPT6L or P5(+)SPT6L(-)P2 in panel D, or significant difference between P5(+)SPT6L and P5(-)SPT6L or P5(+)SPT6L(-)P2 and P5(-)SPT6L(-)P2 in panel E.

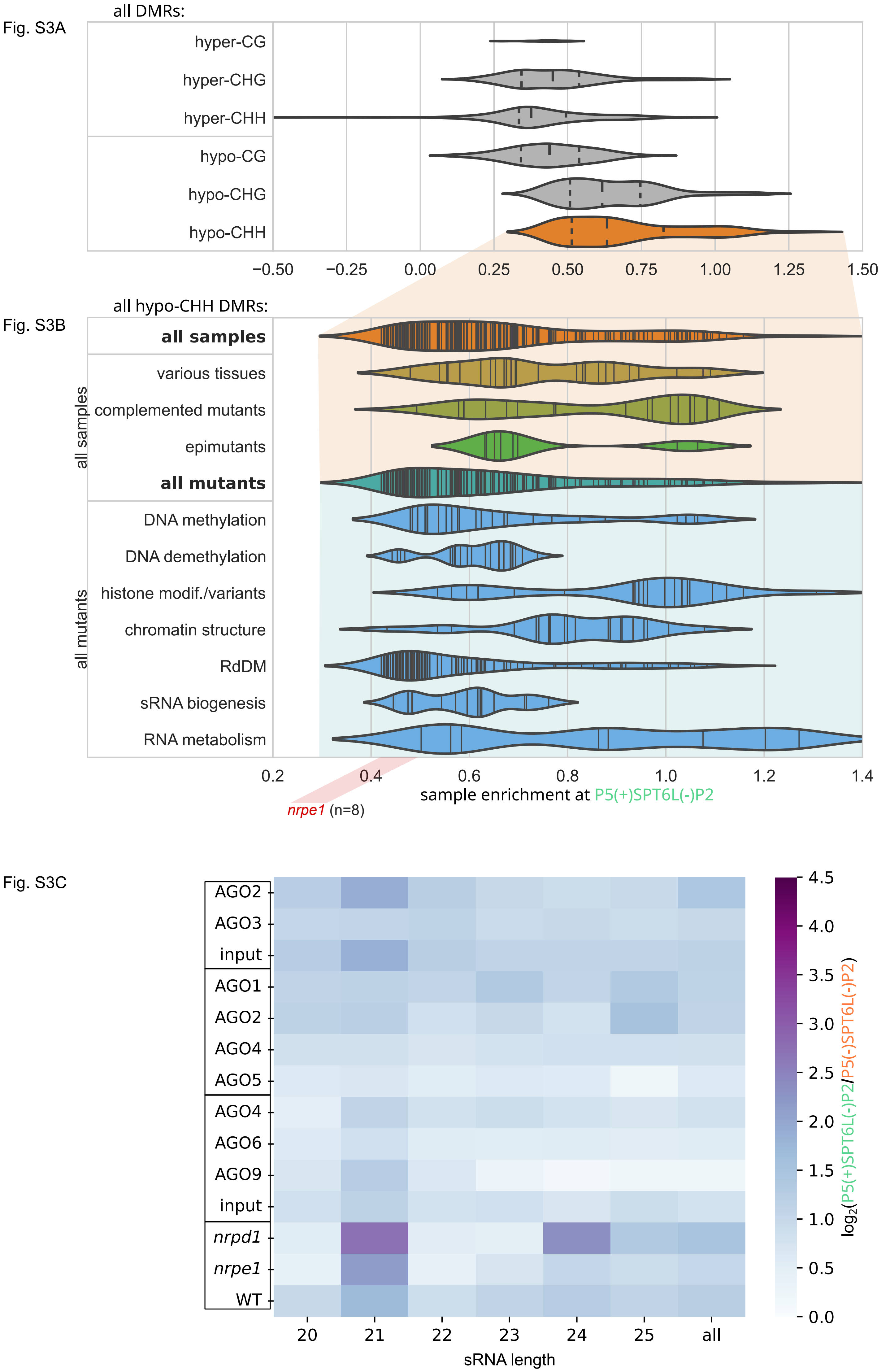
Fig. S3A
all DMRs:
hyper-CG
hyper-CHG
hyper-CHH
hypo-CG
hypo-CHG
hypo-CHH
−0.50
−0.25
0.00
0.25
0.50
0.75
1.00
1.25
1.50
Fig. S3B
all hypo-CHH DMRs:
all samples
various tissues
complemented mutants
epimutants
all mutants
DNA methylation
DNA demethylation
histone modif./variants
chromatin structure
RdDM
sRNA biogenesis
RNA metabolism
all samples
all mutants
0.2
0.4
0.6
0.8
1.0
1.2
1.4
sample enrichment at P5(+)SPT6L(-)P2
nrpe1 (n=8)
Fig. S3C
AGO2
AGO3
input
AGO1
AGO2
AGO4
AGO5
AGO4
AGO6
AGO9
input
nrpd1
nrpe1
WT
20
21
22
23
24
25
all
sRNA length
4.5
4.0
3.5
3.0
2.5
2.0
1.5
1.0
0.5
0.0
log2(P5(+)SPT6L(-)P2/P5(-)SPT6L(-)P2)

Fig. S3D

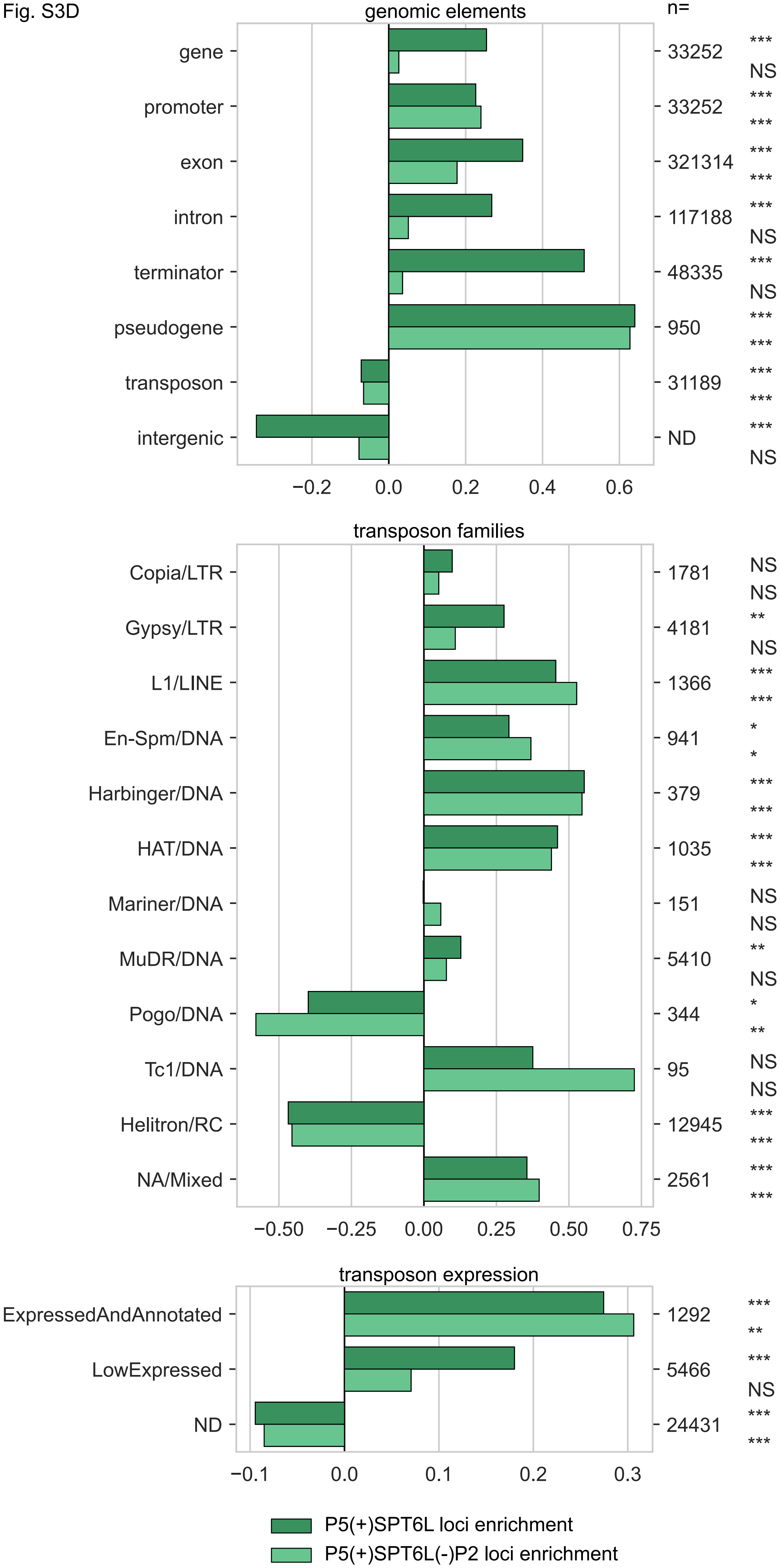

genomic elements
n=
gene
promoter
exon
intron
terminator
pseudogene
transposon
intergenic
33252
33252
321314
117188
48335
950
31189
ND
*** NS
*** ***
*** ***
*** NS
*** NS
*** ***
*** ***
*** NS
−0.2 0.0 0.2 0.4 0.6
transposon families
Copia/LTR
Gypsy/LTR
L1/LINE
En-Spm/DNA
Harbinger/DNA
HAT/DNA
Mariner/DNA
MuDR/DNA
Pogo/DNA
Tc1/DNA
Helitron/RC
NA/Mixed
1781
4181
1366
941
379
1035
151
5410
344
95
12945
2561
NS NS
** NS
*** ***
* *
*** ***
*** ***
NS NS
** NS
* **
NS NS
*** ***
*** ***
−0.50 −0.25 0.00 0.25 0.50 0.75
transposon expression
ExpressedAndAnnotated
LowExpressed
ND
1292
5466
24431
*** **
*** NS
*** ***
−0.1 0.0 0.1 0.2 0.3
P5(+)SPT6L loci enrichment
P5(+)SPT6L(-)P2 loci enrichment

Fig. S3D

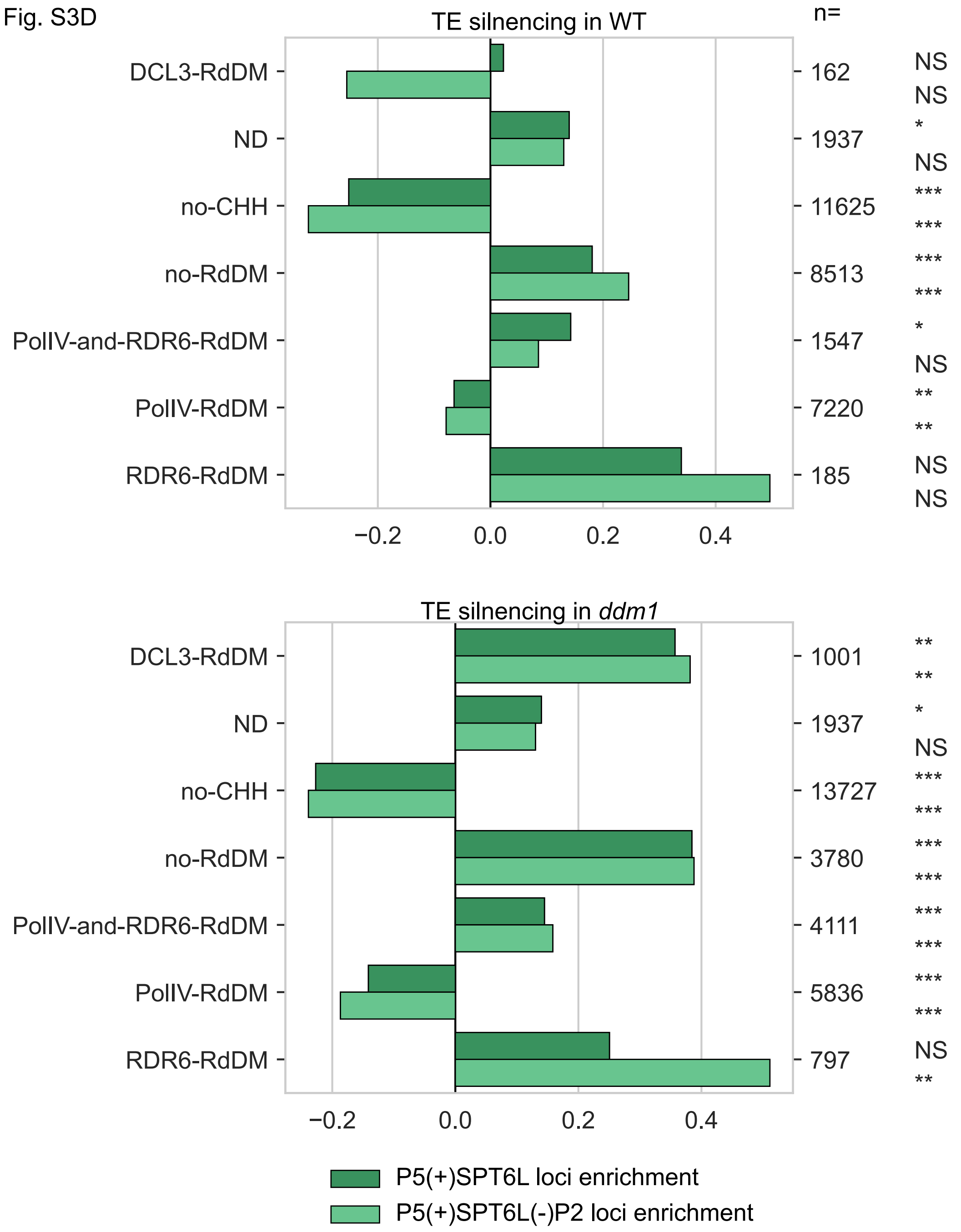

TE silnencing in WT
n=
DCL3-RdDM
ND
no-CHH
no-RdDM
PolIV-and-RDR6-RdDM
PolIV-RdDM
RDR6-RdDM
162
1937
11625
8513
1547
7220
185
NS
NS
*
NS
***
***
***
***
*
NS
**
**
NS
NS
−0.2
0.0
0.2
0.4
TE silnencing in *ddm1*
DCL3-RdDM
ND
no-CHH
no-RdDM
PolIV-and-RDR6-RdDM
PolIV-RdDM
RDR6-RdDM
1001
1937
13727
3780
4111
5836
797
**
**
*
NS
***
***
***
***
***
***
***
***
NS
**
−0.2
0.0
0.2
0.4
P5(+)SPT6L loci enrichment
P5(+)SPT6L(-)P2 loci enrichment

Fig. S3E

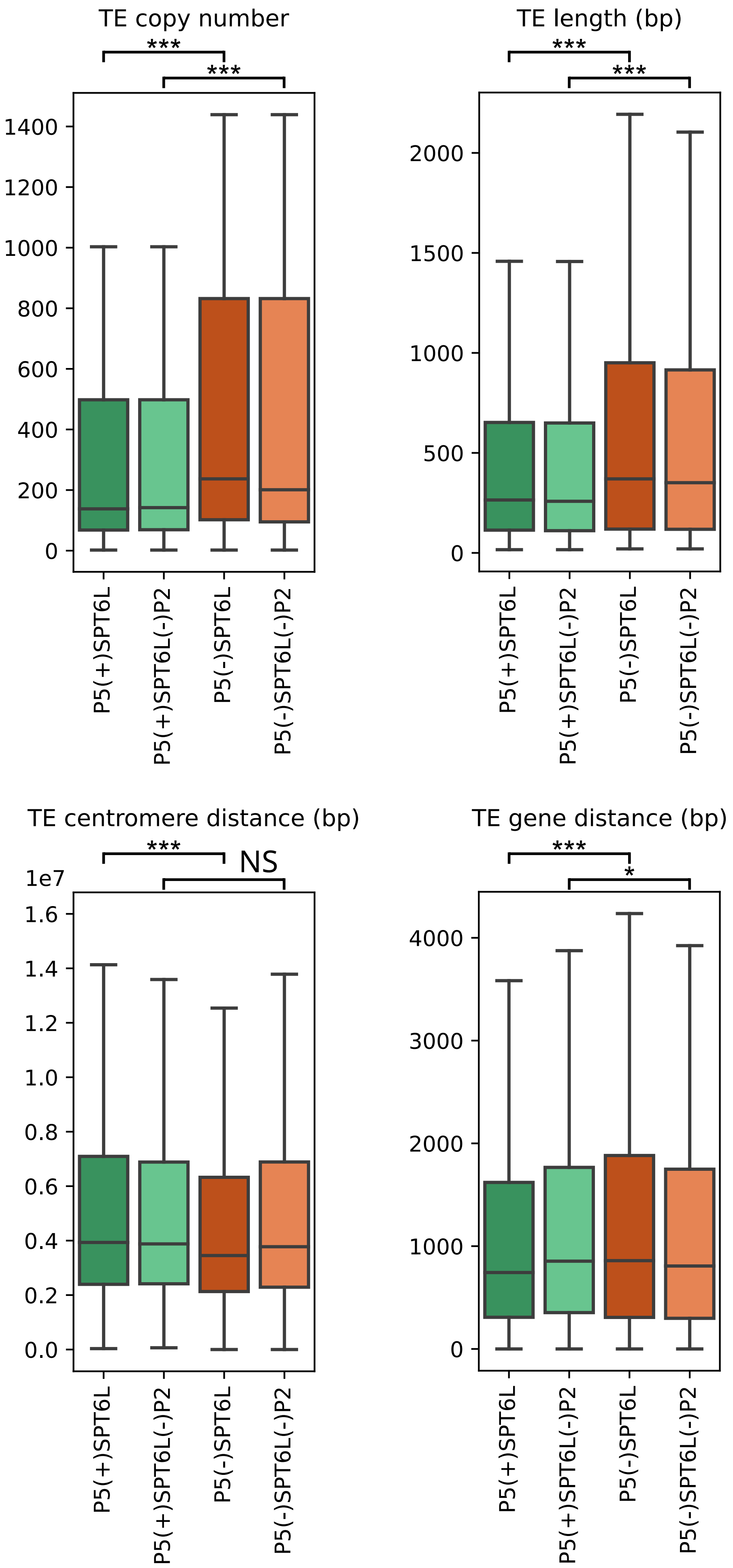
TE copy number
***
***
1400
1200
1000
800
600
400
200
0
P5(+)SPT6L
P5(+)SPT6L(-)P2
P5(-)SPT6L
P5(-)SPT6L(-)P2
TE length (bp)
***
***
2000
1500
1000
500
0
P5(+)SPT6L
P5(+)SPT6L(-)P2
P5(-)SPT6L
P5(-)SPT6L(-)P2
TE centromere distance (bp)
***
NS
1e7
1.6
1.4
1.2
1.0
0.8
0.6
0.4
0.2
0.0
P5(+)SPT6L
P5(+)SPT6L(-)P2
P5(-)SPT6L
P5(-)SPT6L(-)P2
TE gene distance (bp)
***
*
4000
3000
2000
1000
0
P5(+)SPT6L
P5(+)SPT6L(-)P2
P5(-)SPT6L
P5(-)SPT6L(-)P2

Supplemental Fig S4: Evolution of SPT6L, other relevant AGO-hook proteins and DNA methyltransferases in Viridiplantae

(A-G) Rooted maximum-likelihood trees depict the evolution of (A) SPT6/SPT6L, (B) SPT5/SPT5L, (C) NERD, (D) SDE3 and its homologues, (E) largest subunit of RNA polymerases II, IV and V, (F) DNMT3 and DRMs, (G) DNMT1 and CMTs proteins in the *Viridiplantae* clade. Bootstrap values are shown in the branching points of the tree. (A-D) Red coloring depicts proteins that carry an AGO-hook domain (containing at least 3 WG/GW motifs), whereas blue coloring indicates those not possessing this domain. The number of GW/WG motifs indicates the length of this domain. (C) Tree for NERD is unrooted. (E) Yellow coloring depicts the largest subunit of Pol II, blue of Pol IV, light green of Pol V and darker green of Pol IV/V (ancestor of Pols IV and V). The scale bar in a RAxML trees is an estimation of the number of nucleotide substitutions per site. (H-I) Analysis of the C-terminal domain of SPT6(L) in the *Klebsormidium* genus. *Klebsormidium* seems to be the only streptophyte algae that doesn't possess SPT6L with an AGO-hook domain. The C-terminal domain of SPT6(L) in neither *K. flaccidum* nor *K. nitens* did comply with our stringent filter, even though these domains resemble AGO-hook domains in other SPT6L proteins. It remains unclear whether even the truncated AGO-hook-like domain of SPT6(L) in the *Klebsormidium* genus is functional, or if this domain was lost. (H) Sashimi plot of contig kfl00507 of *Klebsormidium nitens* NIES-2285 genome with aligned RNA-seq reads from sequencing project GSM5397554. Reads were aligned with RNA-STAR with default options allowing long introns. There seems to be no introns spanning further than the annotated sequence for SPT6L which rules out the possibility that the domain was originally not found due to alternative splicing. (I) Scheme of CTDs of obtained sequences for SPT6(L) protein from Arabidopsis thaliana and *Klebsormidium nitens*. Colors from blue (low) to red (high) denote the probability of AGO-hook motifs. Remark: when the eukaryotic matrix for W-search is used instead of the plant matrix, then the SPT6(L) passes our criteria as the third tryptophan residue passes our threshold.

Fig. S4A

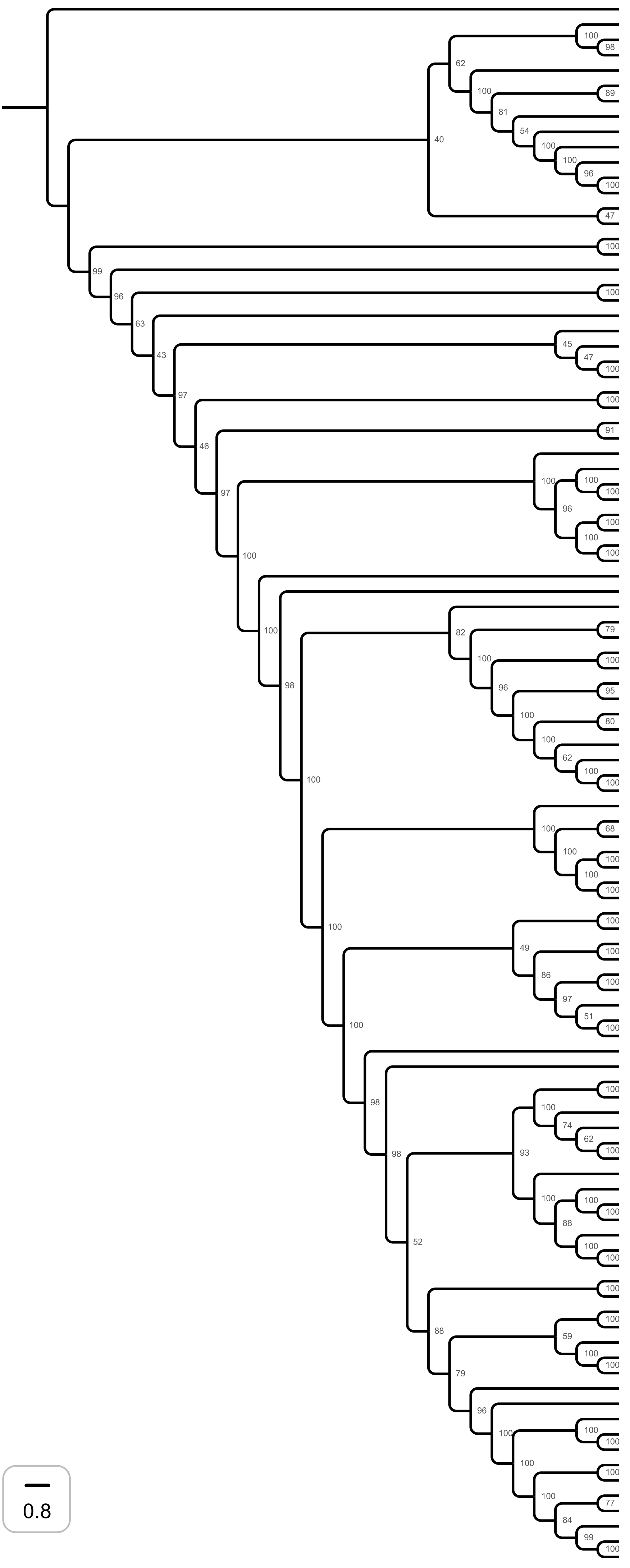

Species	Sequence ID	# GW/WG
Galdieria sulphuraria	XM_005707307.1	0
Micromonas pusilla	XP_003057540.1	1
Ostreococcus lucimarinus	XP_001420894.1	1
Bathycoccus prasinos	XP_007514822.1	0
Ostreobium quekettii	CAD7702599.1	2
Coccomyxa subellipsoidea	C_169_XP_005647292.1	1
Trebouxia sp.	A1_2_KAA6428789.1	0
Auxenochlorella protothecoides	XP_011397843.1	0
Scenedesmus sp.	KAF6260805.1	0
Chlamydomonas reinhardtii	XP_042921444.1	0
Edaphochlamys debaryana	KAG2488784.1	1
Volvox carteri	XP_002958667.1	1
Astrephomene gubernaculifera	GFR40315.1	0
Chlorokybus atmophyticus	GFZF01006451.1	7
Mesostigma viride	jgi|Mesvir1|20970|Mv08037_RA.1	5
Interfilum paradoxum	FPCO_scaffold_2030348	3
Klebsormidium nitens	kfl00507_0030_v1.1	2
Coleochaete scutata	GFXZ01004166.1	4
Mesotaenium kramstae	jgi|Meskra657_3|2180028	3
Mougeotia calospora	GJZN01001526.1	5
Chara braunii	GBG67752.1	26
Anthoceros angustus	MU008166.1	6
Marchantia polymorpha	PTQ43424	34
Physcomitrium patens	XM_024520549.1	9
Physcomitrium patens	XM_024519963.1	11
Selaginella moellendorfii	XP_024519823	10
Selaginella moellendorffii	XM_024664055.1	7
Ceratopteris richardii	KAH7290621	21
Ceratopteris richardii	KAH7314514	14
Cycas revoluta	GBJU01001637.1	16
Cryptomeria japonica	ICQT01009851.1	12
Thuja plicata	Thupl.29382020s0009.1.p	11
Thuja occidentalis	GGJL01009513.1	13
Pinus sylvestris	GILO01692244.1	13
Pinus sylvestris	GILP01759947.1	13
Abies pinsapo	GCZN01015930.1	13
Abies pinsapo	GCZN01015928.1	13
Amborella trichopoda	XM_020672699.1	21
Nymphaea colorata	v1.2|Nycol.A02963.1.p	7
Liriodendron tulipifera	GHTS01012009.1	12
Dendrobium catenatum	XM_020816634.2	13
Asparagus officinalis	XM_020385771.1	6
Musa acuminata	v1|GSMUA_Achr2P00830_001	10
Musa acuminata	v1|GSMUA_Achr11P15640_001	2
Ananas comosus	XM_020244307.1	46
Ananas comosus	XM_020234201.1	4
Brachypodium distachyon	XM_014897374.2	1
Brachypodium distachyon	XM_010232918.3	11
Oryza sativa	XM_015783221.2	12
Zea mays	XM_008651595.2	9
Setaria viridis	XM_034732657.1	15
Panicum hallii	XM_025950289.1	12
Vitis vinifera	XM_010647114.2	17
Theobroma cacao	XM_007010677.2	7
Populus trichocarpa	XM_024593835.1	7
Glycine max	Wm82.a2.v1|Glyma.06G312200.1.p	7
Vigna unguiculata	XM_028063641.1	18
Medicago truncatula	XM_024779411.2	24
Cicer arietinum	XM_004499712.3	24
Spinacia oleracea	Spov3|Spov3_chr4.02535	9
Beta vulgaris	XM_010674818.2	10
Lactuca sativa	V8|Lsat_1_v5_gn_8_83860.1	3
Lactuca sativa	V8|Lsat_1_v5_gn_9_32200.1	24
Nicotiana tabacum	XM_016614012.1	8
Solanum tuberosum	XM_006339186.2	7
Olea europaea	XM_022990656.1	10
Coffea arabica	XM_027215630.1	0
Coffea arabica	XM_027215633.1	12
Vitis vinifera	XM_010655357.2	8
Eucalyptus grandis	XM_039304186.1	8
Prunus persica	XM_020569586.1	2
Malus domestica	v1.1|MD10G1173400	5
Fragaria vesca	XM_011472667.1	9
Prunus persica	XM_020567728.1	8
Malus domestica	v1.1|MD00G1108000	10
Malus domestica	v1.1|MD00G1025500	10
Arachis duranensis	XM_016115189.2	10
Vigna unguiculata	XM_028069878.1	8
Glycine max	Wm82.a2.v1|Glyma.19G125600.1.p	8
Glycine max	Wm82.a2.v1|Glyma.03G121000.1.p	9
Cicer arietinum	XM_004493257.3	7
Medicago truncatula	XM_039827554.1	11
Medicago truncatula	XM_039827995.1	1
Citrus clementina	XM_024182253.1	7
Citrus clementina	v1.0|Ciclev10013566m	7
Populus trichocarpa	XM_002322561.3	11
Populus trichocarpa	XM_024604608.1	7
Theobroma cacao	XM_007010649.2	7
Gossypium raimondii	XM_012599158.1	2
Gossypium raimondii	XM_012593495.1	9
Carica papaya	XM_022035839.1	2
Tarenaya hassleriana	XP_010528633.1	9
Capsella rubella	XM_023788783.1	0
Arabidopsis thaliana	NM_105000.1	0
Capsella rubella	XM_023788580.1	0
Capsella rubella	XM_006300582.2	13
Arabidopsis thaliana	NM_105218.5	12
Eutrema salsugineum	XM_024156252.1	11
Eutrema salsugineum	XM_024150584.1	2
Brassica rapa	v1.3|Brara.B01624.1.p	8
Brassica oleracea	XM_013736047.1	9
Brassica rapa	v1.3|Brara.G02597.1.p	8

Fig. S4B

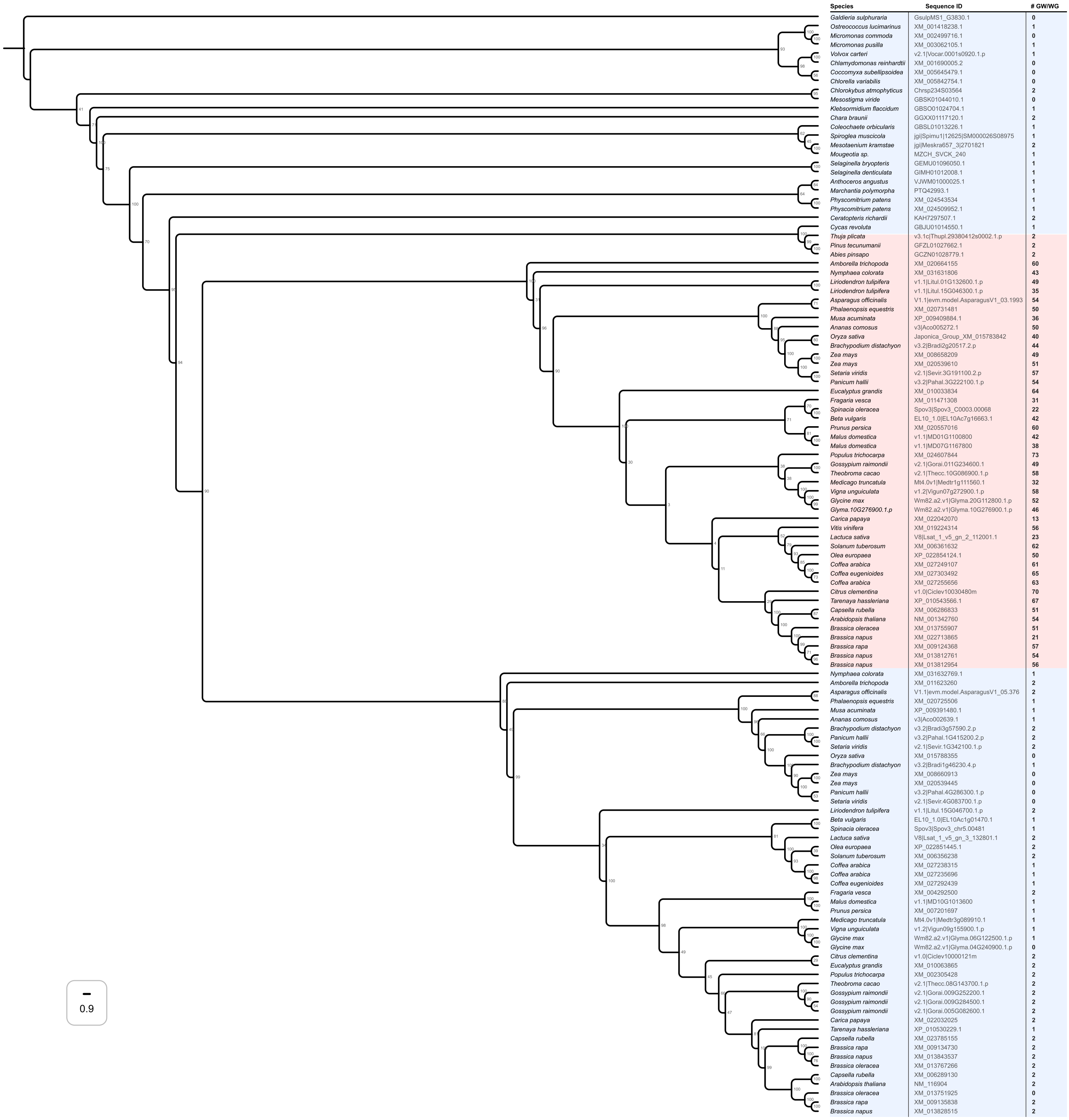
Species | Sequence ID | # GW/WG
Galdieria sulphuraria | GsulpMS1_G3830.1 | 0
Ostreococcus lucimarinus | XM_001418238.1 | 1
Micromonas commoda | XM_002499716.1 | 0
Micromonas pusilla | XM_003062105.1 | 1
Volvox carteri | v2.1|Vocar.0001s0920.1.p | 1
Chlamydomonas reinhardtii | XM_001690005.2 | 0
Coccomyxa subellipsoidea | XM_005645479.1 | 0
Chlorella variabilis | XM_005842754.1 | 0
Chlorokybus atmophyticus | Chrsp234S03564 | 2
Mesostigma viride | GBSK01044010.1 | 0
Klebsormidium flaccidum | GBSO01024704.1 | 1
Chara braunii | GGXX01117120.1 | 2
Coleochaete orbicularis | GBSL01013226.1 | 1
Spiroglea muscicola | jgi|Spimu1|12625|SM000026S08975 | 1
Mesotaenium kramstae | jgi|Meskra657_3|2701821 | 2
Mougeotia sp. | MZCH_SVCK_240 | 1
Selaginella bryopteris | GEMU01096050.1 | 1
Selaginella denticulata | GIMH01012008.1 | 1
Anthoceros angustus | VJWM01000025.1 | 1
Marchantia polymorpha | PTQ42993.1 | 1
Physcomitrium patens | XM_024543534 | 1
Physcomitrium patens | XM_024509952.1 | 1
Ceratopteris richardii | KAH7297507.1 | 2
Cycas revoluta | GBJU01014550.1 | 1
Thuja plicata | v3.1c|Thupl.29380412s0002.1.p | 2
Pinus tecunumanii | GFZL01027662.1 | 2
Abies pinsapo | GCZN01028779.1 | 2
Amborella trichopoda | XM_020664155 | 60
Nymphaea colorata | XM_031631806 | 43
Liriodendron tulipifera | v1.1|Litul.01G132600.1.p | 49
Liriodendron tulipifera | v1.1|Litul.15G046300.1.p | 35
Asparagus officinalis | V1.1|evm.model.AsparagusV1_03.1993 | 54
Phalaenopsis equestris | XM_020731481 | 50
Musa acuminata | XP_009409884.1 | 36
Ananas comosus | v3|Aco005272.1 | 50
Oryza sativa | Japonica_Group_XM_015783842 | 40
Brachypodium distachyon | v3.2|Bradi2g20517.2.p | 44
Zea mays | XM_008658209 | 49
Zea mays | XM_020539610 | 51
Setaria viridis | v2.1|Sevir.3G191100.2.p | 57
Panicum hallii | v3.2|Pahal.3G222100.1.p | 54
Eucalyptus grandis | XM_010033834 | 64
Fragaria vesca | XM_011471308 | 31
Spinacia oleracea | Spov3|Spov3_C0003.00068 | 22
Beta vulgaris | EL10_1.0|EL10Ac7g16663.1 | 42
Prunus persica | XM_020557016 | 60
Malus domestica | v1.1|MD01G1100800 | 42
Malus domestica | v1.1|MD07G1167800 | 38
Populus trichocarpa | XM_024607844 | 73
Gossypium raimondii | v2.1|Gorai.011G234600.1 | 49
Theobroma cacao | v2.1|Thecc.10G086900.1.p | 58
Medicago truncatula | Mt4.0v1|Medtr1g111560.1 | 32
Vigna unguiculata | v1.2|Vigun07g272900.1.p | 58
Glycine max | Wm82.a2.v1|Glyma.20G112800.1.p | 52
Glyma.10G276900.1.p | Wm82.a2.v1|Glyma.10G276900.1.p | 46
Carica papaya | XM_022042070 | 13
Vitis vinifera | XM_019224314 | 56
Lactuca sativa | V8|Lsat_1_v5_gn_2_112001.1 | 23
Solanum tuberosum | XM_006361632 | 62
Olea europaea | XP_022854124.1 | 50
Coffea arabica | XM_027249107 | 61
Coffea eugenioides | XM_027303492 | 65
Coffea arabica | XM_027255656 | 63
Citrus clementina | v1.0|Ciclev10030480m | 70
Tarenaya hassleriana | XP_010543566.1 | 67
Capsella rubella | XM_006286833 | 51
Arabidopsis thaliana | NM_001342760 | 54
Brassica oleracea | XM_013755907 | 51
Brassica napus | XM_022713865 | 21
Brassica rapa | XM_009124368 | 57
Brassica napus | XM_013812761 | 54
Brassica napus | XM_013812954 | 56
Nymphaea colorata | XM_031632769.1 | 1
Amborella trichopoda | XM_011623260 | 2
Asparagus officinalis | V1.1|evm.model.AsparagusV1_05.376 | 2
Phalaenopsis equestris | XM_020725506 | 1
Musa acuminata | XP_009391480.1 | 1
Ananas comosus | v3|Aco002639.1 | 1
Brachypodium distachyon | v3.2|Bradi3g57590.2.p | 2
Panicum hallii | v3.2|Pahal.1G415200.2.p | 2
Setaria viridis | v2.1|Sevir.1G342100.1.p | 2
Oryza sativa | XM_015788355 | 0
Brachypodium distachyon | v3.2|Bradi1g46230.4.p | 1
Zea mays | XM_008660913 | 0
Zea mays | XM_020539445 | 0
Panicum hallii | v3.2|Pahal.4G286300.1.p | 0
Setaria viridis | v2.1|Sevir.4G083700.1.p | 0
Liriodendron tulipifera | v1.1|Litul.15G046700.1.p | 2
Beta vulgaris | EL10_1.0|EL10Ac1g01470.1 | 1
Spinacia oleracea | Spov3|Spov3_chr5.00481 | 1
Lactuca sativa | V8|Lsat_1_v5_gn_3_132801.1 | 2
Olea europaea | XP_022851445.1 | 2
Solanum tuberosum | XM_006356238 | 2
Coffea arabica | XM_027238315 | 1
Coffea arabica | XM_027235696 | 1
Coffea eugenioides | XM_027292439 | 1
Fragaria vesca | XM_004292500 | 2
Malus domestica | v1.1|MD10G1013600 | 1
Prunus persica | XM_007201697 | 1
Medicago truncatula | Mt4.0v1|Medtr3g089910.1 | 1
Vigna unguiculata | v1.2|Vigun09g155900.1.p | 1
Glycine max | Wm82.a2.v1|Glyma.06G122500.1.p | 1
Glycine max | Wm82.a2.v1|Glyma.04G240900.1.p | 0
Citrus clementina | v1.0|Ciclev10000121m | 2
Eucalyptus grandis | XM_010063865 | 2
Populus trichocarpa | XM_002305428 | 2
Theobroma cacao | v2.1|Thecc.08G143700.1.p | 2
Gossypium raimondii | v2.1|Gorai.009G252200.1 | 2
Gossypium raimondii | v2.1|Gorai.009G284500.1 | 2
Gossypium raimondii | v2.1|Gorai.005G082600.1 | 2
Carica papaya | XM_022032025 | 2
Tarenaya hassleriana | XP_010530229.1 | 1
Capsella rubella | XM_023785155 | 2
Brassica rapa | XM_009134730 | 2
Brassica napus | XM_013843537 | 2
Brassica oleracea | XM_013767266 | 2
Capsella rubella | XM_006289130 | 2
Arabidopsis thaliana | NM_116904 | 2
Brassica oleracea | XM_013751925 | 0
Brassica rapa | XM_009135838 | 2
Brassica napus | XM_013828515 | 2
0.9

Fig. S4C

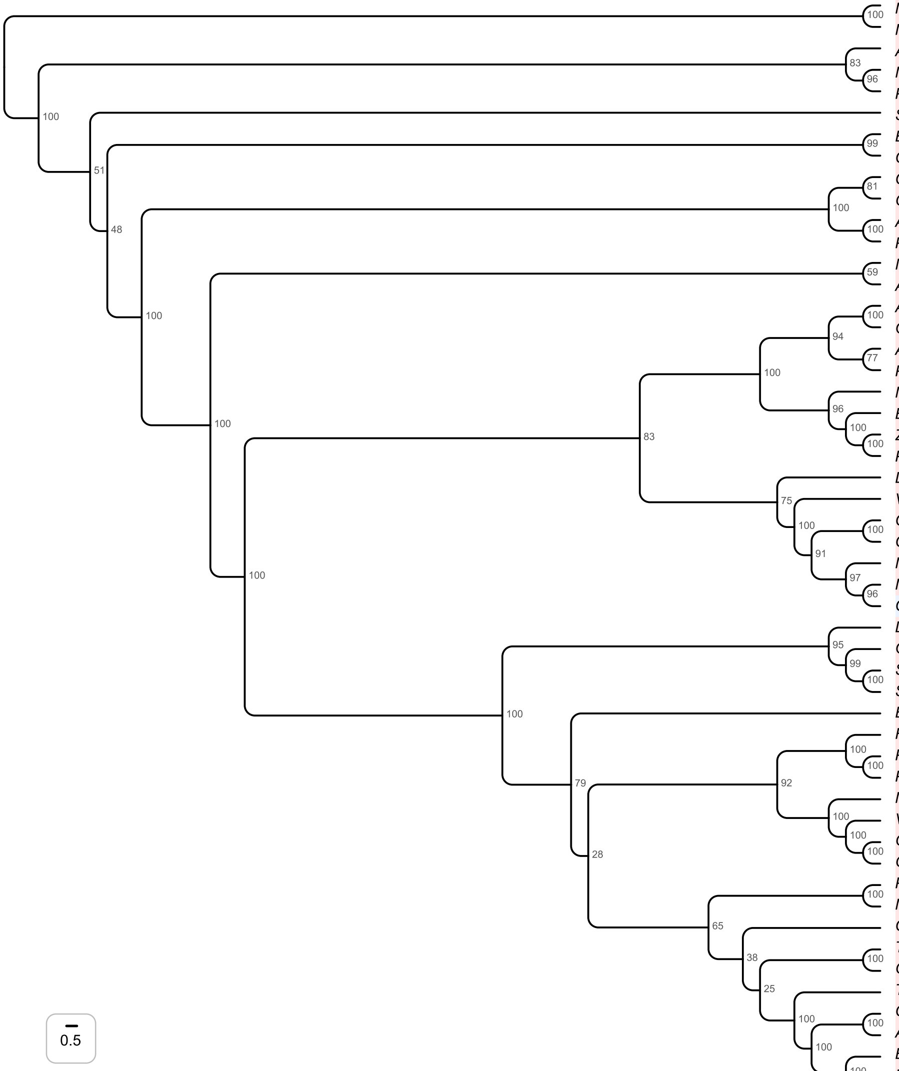

Species	Sequence ID	# GW/WG
Mesotaenium endlicherianum	Mesen1|5796|ME000293S04950	5
Mesotaenium kramstae	Meskra657_3|204237|CE203456_10532	4
Anthoceros angustus	MU008185.1	4
Marchantia polymorpha	GEFO01024060.1	5
Physcomitrium patens	XM_024540864.1	6
Selaginella bryopteris	GEMU01103855.1	4
Brainea insignis	GFUE01030576.1	4
Ceratopteris richardii	GIXU01028817.1	6
Cycas revoluta	GGZS01014446.1	16
Ginkgo biloba	GGBW01011546.1	24
Abies pinsapo	GCZN01027697.1	17
Pinus sylvestris	GHKX01012973.1	18
Nymphaea colorata	XM_031627670.1	4
Amborella trichopoda	XM_011626888.2	17
Ananas comosus	XP_020093697.1	6
Oryza sativa	XM_015758901.2	3
Asparagus officinalis	V1.1|AsparagusV1_Unassigned.582	11
Phalaenopsis equestris	XM_020727459.1	7
Musa acuminata	XP_009395393.1	3
Brachypodium distachyon	v3.1|Bradi4g35977.3.p	4
Zea mays	XM_008659006.4	6
Panicum hallii	v3.2|Pahal.5G162300.1.p	6
Liriodendron tulipifera	v1.1|Litul.04G072000.1.p	16
Vitis vinifera	v2.1|VIT_204s0023g02950.2	8
Coffea arabica	XM_027221728.1	3
Coffea arabica	XM_027217781.1	3
Malus domestica	XM_008346037.3	3
Manihot esculenta	XM_021749076.2	3
Carica papaya	XM_022052612.1	0
Lactuca sativa	V8|Lsat_1_v5_gn_8_128721.1	6
Olea europaea	XM_023040276.1	8
Solanum lycopersicum	XM_010314914.3	3
Solanum tuberosum	XM_006356320.2	3
Eucalyptus grandis	XM_010067912.3	7
Fragaria vesca	XM_004292388.2	11
Prunus persica	v2.1|Prupe.8G085700.1.p	9
Prunus persica	XP_020409470.1	9
Medicago truncatula	XM_024779664.2	5
Vigna unguiculata	v1.2|Vigun09g269600.1.p	5
Glycine max	Wm82.a4.v1|Glyma.06G008300.1.p	5
Glycine soja	XM_028378725.1	5
Populus alba	XM_035071778.1	11
Manihot esculenta	XM_021755439.2	7
Citrus clementina	XM_006423406.2	5
Theobroma cacao	v2.1|Thecc.02G076000.1.p	9
Gossypium raimondii	XM_012624939.1	5
Tarenaya hassleriana	XP_019056896.1	5
Capsella rubella	XM_006296755.2	6
Arabidopsis thaliana	NM_127202.5	6
Brassica oleracea	v1.0|Bol012694	6
Brassica rapa	v1.3|Brara.I00996.1.p	6
Brassica napus	XM_013800386.2	6

Fig. S4D

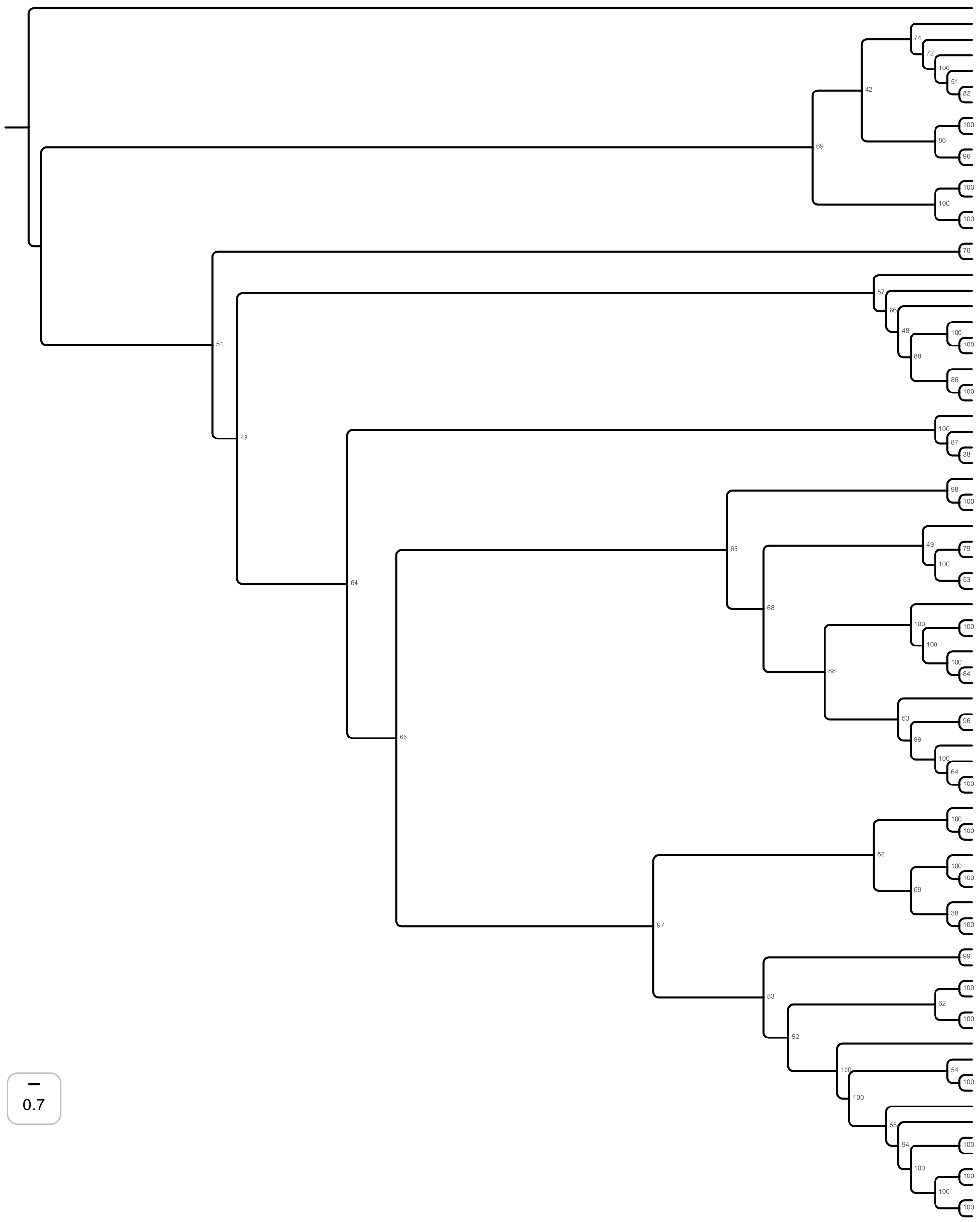

Species	Sequence ID	# GW/WG
Galdieria sulphuraria	Gsulp5572_G6141.1_UPF1	1
Klebsormidium flaccidum	kfl00006_0480_v1.1	0
Klebsormidium flaccidum	kfl00077_0020_v1.1	0
Spiroglea muscicola	jgi|Spimu1|23425	0
Mesotaenium kramstae	jgi|Meskra657_3|2582996	0
Klebsormidium flaccidum	kfl00120_0100_v1.1	0
Chara braunii	CHBRA412g00140	1
Sellaginella moellendorffii	v1.0|236589	0
Sellaginella moellendorffii	v1.0|430895	0
Botrychium lunaria	GIJZ01000402.1	0
Physcomitrella patens	Pp3c12_8920V3.1	0
Thuja occidentalis	GGJL01020185.1	0
Thuja plicata	v3.1c|Thupl.29380709s0004.1.p	1
Pinus lambertiana	GEUZ01002387.1	0
Pinus flexilis	GHWE01188158.1	0
Amborella trichopoda	XM_020664199.1	0
Nymphaea colorata	XM_031619418.1	0
Liriodendron tulipifera	v1.1|Litul.16G005300.1.p	0
Asparagus officinalis	V1.1|AsparagusV1_08.1887	0
Phalaenopsis equestris	XM_020734797.1	0
Musa acuminata	v1|GSMUA_Achr3P25460_001	1
Zingiber officinale	XM_042617426.1	1
Zingiber officinale	XM_042612330.1	1
Ananas comosus	v3|Aco000226.1	1
Zea mays	XM_008653011.3	0
Oryza sativa	XM_015772278.2	0
Carica papaya	XM_022035582.1	0
Populus trichocarpa	XM_006382673.2	0
Eucalyptus grandis	XM_039300975.1	0
Theobroma cacao	v2.1|Thecc.05G291200.1.p	1
Camellia sinensis	XM_028224285.1	0
Impatiens glandulifera	XM_047477804.1	0
Impatiens glandulifera	XM_047461405.1	0
Daucus carota	XM_017400410.1	3
Cynara cardunculus	XM_025112441.1	4
Lactuca sativa	XM_023902859.2	7
Erigeron canadensis	XM_043763207.1	5
Helianthus annuus	XM_022154715.2	3
Olea europaea	v1.0|Oeu018974.1	1
Salvia hispanica	XM_048096010.1	3
Salvia splendens	XM_042200070.1	2
Salvia hispanica	XM_048127576.1	2
Salvia splendens	XM_042182159.1	3
Salvia splendens	XM_042209340.1	3
Coffea arabica	XM_027261613.1	0
Solanum lycopersicum	XM_004240994.4	1
Capsicum annuum	XM_016719860.2	6
Nicotiana attenuata	XM_019410890.1	4
Nicotiana tabacum	XM_016647526.1	2
Nicotiana tabacum	XM_016608630.1	3
Nicotiana tomentosiformis	XM_009619903.3	3
Glycine soja	XM_028324683.1	1
Medicago truncatula	XM_003595558.4	2
Medicago truncatula	XM_003610728.3	2
Fragaria vesca	XM_004297068.2	0
Prunus persica	XM_020564494.1	1
Malus domestica	v1.1|MD13G1196500	0
Morus notabilis	XM_010110024.2	1
Ziziphus jujuba	XM_016032673.2	1
Ziziphus jujuba	XM_025069116.1	1
Manihot esculenta	XM_021751855.2	0
Populus trichocarpa	XM_006379784.2	0
Gossypium raimondii	XM_012631726.1	0
Gossypium hirsutum	XM_041101358.1	0
Citrus clementina	XM_024179974.1	2
Citrus sinensis	XM_006489365.2	2
Tarenaya hassleriana	XM_010523987.2	0
Capsella rubella	XM_006306592.2	4
Camelina sativa	XM_010486686.2	5
Camelina sativa	XM_010482052.1	2
Arabidopsis thaliana	NM_100425.4	5
Eutrema salsugineum	XM_006417961.2	7
Raphanus sativus	XM_018610486.1	0
Raphanus sativus	XM_018580283.1	9
Brassica oleracea	XM_013730784.1	5
Brassica napus	XM_022702907.1	5
Brassica rapa	XM_009120906.3	4
Brassica napus	XM_022693149.1	4

Fig. S4E

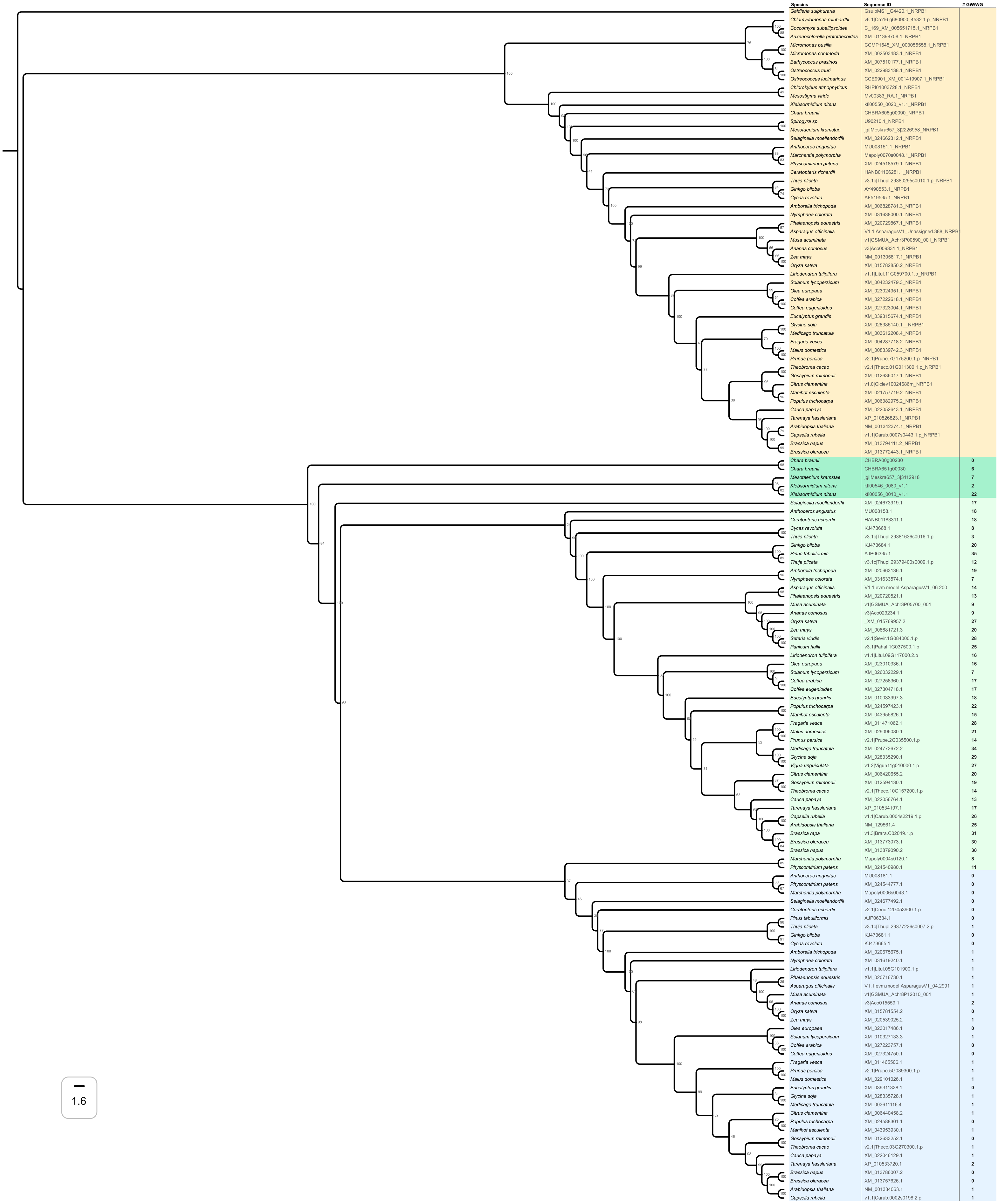
Species Sequence ID # GW/WG
Galdieria sulphuraria GsulpMS1_G4420.1_NRPB1
Chlamydomonas reinhardtii v6.1|Cre16.g680900_4532.1.p_NRPB1
Coccomyxa subellipsoidea C_169_XM_005651715.1_NRPB1
Auxenochlorella protothecoides XM_011398708.1_NRPB1
Micromonas pusilla CCMP1545_XM_003055558.1_NRPB1
Micromonas commoda XM_002503483.1_NRPB1
Bathycoccus prasinos XM_007510177.1_NRPB1
Ostreococcus tauri XM_022983138.1_NRPB1
Ostreococcus lucimarinus CCE9901_XM_001419907.1_NRPB1
Chlorokybus atmophyticus RHPI01003728.1_NRPB1
Mesostigma viride Mv00383_RA.1_NRPB1
Klebsormidium nitens kfl00550_0020_v1.1_NRPB1
Chara braunii CHBRA608g00090_NRPB1
Spirogyra sp. U90210.1_NRPB1
Mesotaenium kramstae jgi|Meskra657_3|2226958_NRPB1
Selaginella moellendorffii XM_024662312.1_NRPB1
Anthoceros angustus MU008151.1_NRPB1
Marchantia polymorpha Mapoly0070s0048.1_NRPB1
Physcomitrium patens XM_024518579.1_NRPB1
Ceratopteris richardii HANB01166281.1_NRPB1
Thuja plicata v3.1c|Thupl.29380295s0010.1.p_NRPB1
Ginkgo biloba AY490553.1_NRPB1
Cycas revoluta AF519535.1_NRPB1
Amborella trichopoda XM_006828781.3_NRPB1
Nymphaea colorata XM_031638000.1_NRPB1
Phalaenopsis equestris XM_020729867.1_NRPB1
Asparagus officinalis V1.1|AsparagusV1_Unassigned.388_NRPB1
Musa acuminata v1|GSMUA_Achr3P00590_001_NRPB1
Ananas comosus v3|Aco009331.1_NRPB1
Zea mays NM_001305817.1_NRPB1
Oryza sativa XM_015782850.2_NRPB1
Liriodendron tulipifera v1.1|Litul.11G059700.1.p_NRPB1
Solanum lycopersicum XM_004232479.3_NRPB1
Olea europaea XM_023024951.1_NRPB1
Coffea arabica XM_027222618.1_NRPB1
Coffea eugenioides XM_027323004.1_NRPB1
Eucalyptus grandis XM_039315674.1_NRPB1
Glycine soja XM_028385140.1__NRPB1
Medicago truncatula XM_003612208.4_NRPB1
Fragaria vesca XM_004287718.2_NRPB1
Malus domestica XM_008339742.3_NRPB1
Prunus persica v2.1|Prupe.7G175200.1.p_NRPB1
Theobroma cacao v2.1|Thecc.01G011300.1.p_NRPB1
Gossypium raimondii XM_012636017.1_NRPB1
Citrus clementina v1.0|Ciclev10024686m_NRPB1
Manihot esculenta XM_021757719.2_NRPB1
Populus trichocarpa XM_006382975.2_NRPB1
Carica papaya XM_022052643.1_NRPB1
Tarenaya hassleriana XP_010526823.1_NRPB1
Arabidopsis thaliana NM_001342374.1_NRPB1
Capsella rubella v1.1|Carub.0007s0443.1.p_NRPB1
Brassica napus XM_013794111.2_NRPB1
Brassica oleracea XM_013772443.1_NRPB1
Chara braunii CHBRA00g00230 0
Chara braunii CHBRA651g00030 6
Mesotaenium kramstae jgi|Meskra657_3|3112918 7
Klebsormidium nitens kfl00546_0080_v1.1 2
Klebsormidium nitens kfl00056_0010_v1.1 22
Selaginella moellendorffii XM_024673919.1 17
Anthoceros angustus MU008158.1 18
Ceratopteris richardii HANB01183311.1 18
Cycas revoluta KJ473668.1 8
Thuja plicata v3.1c|Thupl.29381636s0016.1.p 3
Ginkgo biloba KJ473684.1 20
Pinus tabuliformis AJP06335.1 35
Thuja plicata v3.1c|Thupl.29379400s0009.1.p 12
Amborella trichopoda XM_020663136.1 19
Nymphaea colorata XM_031633574.1 7
Asparagus officinalis V1.1|evm.model.AsparagusV1_06.200 14
Phalaenopsis equestris XM_020720521.1 13
Musa acuminata v1|GSMUA_Achr3P05700_001 9
Ananas comosus v3|Aco023234.1 9
Oryza sativa _XM_015769957.2 27
Zea mays XM_008681721.3 20
Setaria viridis v2.1|Sevir.1G084000.1.p 28
Panicum hallii v3.1|Pahal.1G037500.1.p 25
Liriodendron tulipifera v1.1|Litul.09G117000.2.p 16
Olea europaea XM_023010336.1 16
Solanum lycopersicum XM_026032229.1 7
Coffea arabica XM_027258360.1 17
Coffea eugenioides XM_027304718.1 17
Eucalyptus grandis XM_010033997.3 18
Populus trichocarpa XM_024597423.1 22
Manihot esculenta XM_043955826.1 15
Fragaria vesca XM_011471062.1 28
Malus domestica XM_029096080.1 21
Prunus persica v2.1|Prupe.2G035500.1.p 14
Medicago truncatula XM_024772672.2 34
Glycine soja XM_028335290.1 29
Vigna unguiculata v1.2|Vigun11g010000.1.p 27
Citrus clementina XM_006420655.2 20
Gossypium raimondii XM_012594130.1 19
Theobroma cacao v2.1|Thecc.10G157200.1.p 14
Carica papaya XM_022056764.1 13
Tarenaya hassleriana XP_010534197.1 17
Capsella rubella v1.1|Carub.0004s2219.1.p 26
Arabidopsis thaliana NM_129561.4 25
Brassica rapa v1.3|Brara.C02049.1.p 31
Brassica oleracea XM_013773073.1 30
Brassica napus XM_013879090.2 30
Marchantia polymorpha Mapoly0004s0120.1 8
Physcomitrium patens XM_024540980.1 11
Anthoceros angustus MU008181.1 0
Physcomitrium patens XM_024544777.1 0
Marchantia polymorpha Mapoly0006s0043.1 0
Selaginella moellendorffii XM_024677492.1 0
Ceratopteris richardii v2.1|Ceric.12G053900.1.p 0
Pinus tabuliformis AJP06334.1 0
Thuja plicata v3.1c|Thupl.29377226s0007.2.p 1
Ginkgo biloba KJ473681.1 0
Cycas revoluta KJ473665.1 0
Amborella trichopoda XM_020675675.1 1
Nymphaea colorata XM_031619240.1 1
Liriodendron tulipifera v1.1|Litul.05G101900.1.p 1
Phalaenopsis equestris XM_020716730.1 1
Asparagus officinalis V1.1|evm.model.AsparagusV1_04.2991 1
Musa acuminata v1|GSMUA_Achr8P12010_001 1
Ananas comosus v3|Aco015559.1 2
Oryza sativa XM_015781554.2 0
Zea mays XM_020539025.2 1
Olea europaea XM_023017486.1 0
Solanum lycopersicum XM_010327133.3 1
Coffea arabica XM_027223757.1 0
Coffea eugenioides XM_027324750.1 0
Fragaria vesca XM_011465506.1 1
Prunus persica v2.1|Prupe.5G089300.1.p 1
Malus domestica XM_029101026.1 1
Eucalyptus grandis XM_039311328.1 0
Glycine soja XM_028335728.1 1
Medicago truncatula XM_003611116.4 1
Citrus clementina XM_006440458.2 1
Populus trichocarpa XM_024588301.1 0
Manihot esculenta XM_043953930.1 1
Gossypium raimondii XM_012633252.1 0
Theobroma cacao v2.1|Thecc.03G270300.1.p 1
Carica papaya XM_022046129.1 1
Tarenaya hassleriana XP_010533720.1 2
Brassica napus XM_013786007.2 0
Brassica oleracea XM_013757626.1 0
Arabidopsis thaliana NM_001334063.1 1
Capsella rubella v1.1|Carub.0002s0198.2.p 1
1.6

Fig. S4F

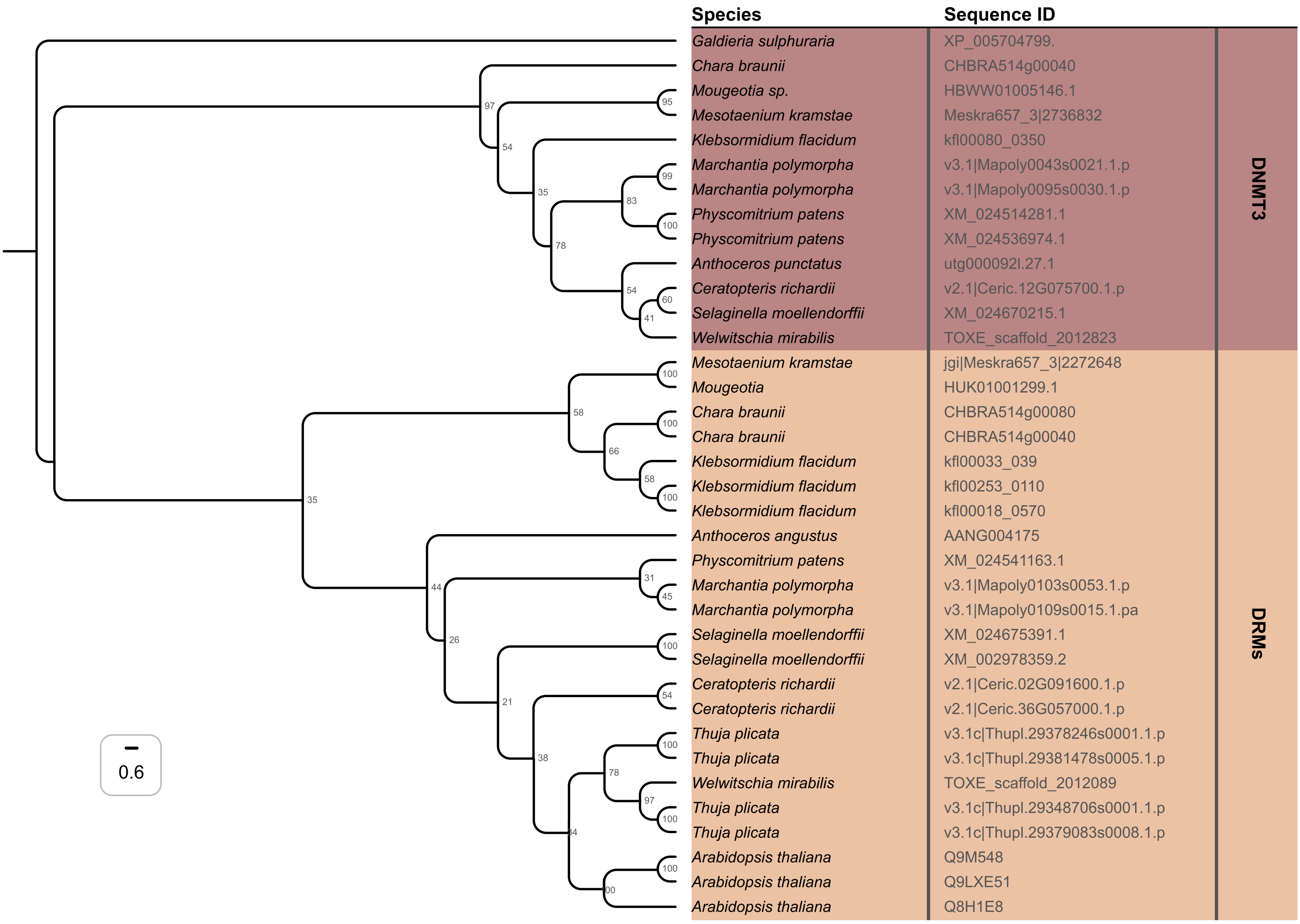
Species
Sequence ID
Galdieria sulphuraria XP_005704799.
Chara braunii CHBRA514g00040
Mougeotia sp. HBWW01005146.1
Mesotaenium kramstae Meskra657_3|2736832
Klebsormidium flacidum kfl00080_0350
Marchantia polymorpha v3.1|Mapoly0043s0021.1.p
Marchantia polymorpha v3.1|Mapoly0095s0030.1.p
Physcomitrium patens XM_024514281.1
Physcomitrium patens XM_024536974.1
Anthoceros punctatus utg000092l.27.1
Ceratopteris richardii v2.1|Ceric.12G075700.1.p
Selaginella moellendorffii XM_024670215.1
Welwitschia mirabilis TOXE_scaffold_2012823
Mesotaenium kramstae jgi|Meskra657_3|2272648
Mougeotia HUK01001299.1
Chara braunii CHBRA514g00080
Chara braunii CHBRA514g00040
Klebsormidium flacidum kfl00033_039
Klebsormidium flacidum kfl00253_0110
Klebsormidium flacidum kfl00018_0570
Anthoceros angustus AANG004175
Physcomitrium patens XM_024541163.1
Marchantia polymorpha v3.1|Mapoly0103s0053.1.p
Marchantia polymorpha v3.1|Mapoly0109s0015.1.pa
Selaginella moellendorffii XM_024675391.1
Selaginella moellendorffii XM_002978359.2
Ceratopteris richardii v2.1|Ceric.02G091600.1.p
Ceratopteris richardii v2.1|Ceric.36G057000.1.p
Thuja plicata v3.1c|Thupl.29378246s0001.1.p
Thuja plicata v3.1c|Thupl.29381478s0005.1.p
Welwitschia mirabilis TOXE_scaffold_2012089
Thuja plicata v3.1c|Thupl.29348706s0001.1.p
Thuja plicata v3.1c|Thupl.29379083s0008.1.p
Arabidopsis thaliana Q9M548
Arabidopsis thaliana Q9LXE51
Arabidopsis thaliana Q8H1E8
DNMT3
DRMs
0.6

Fig. S4G

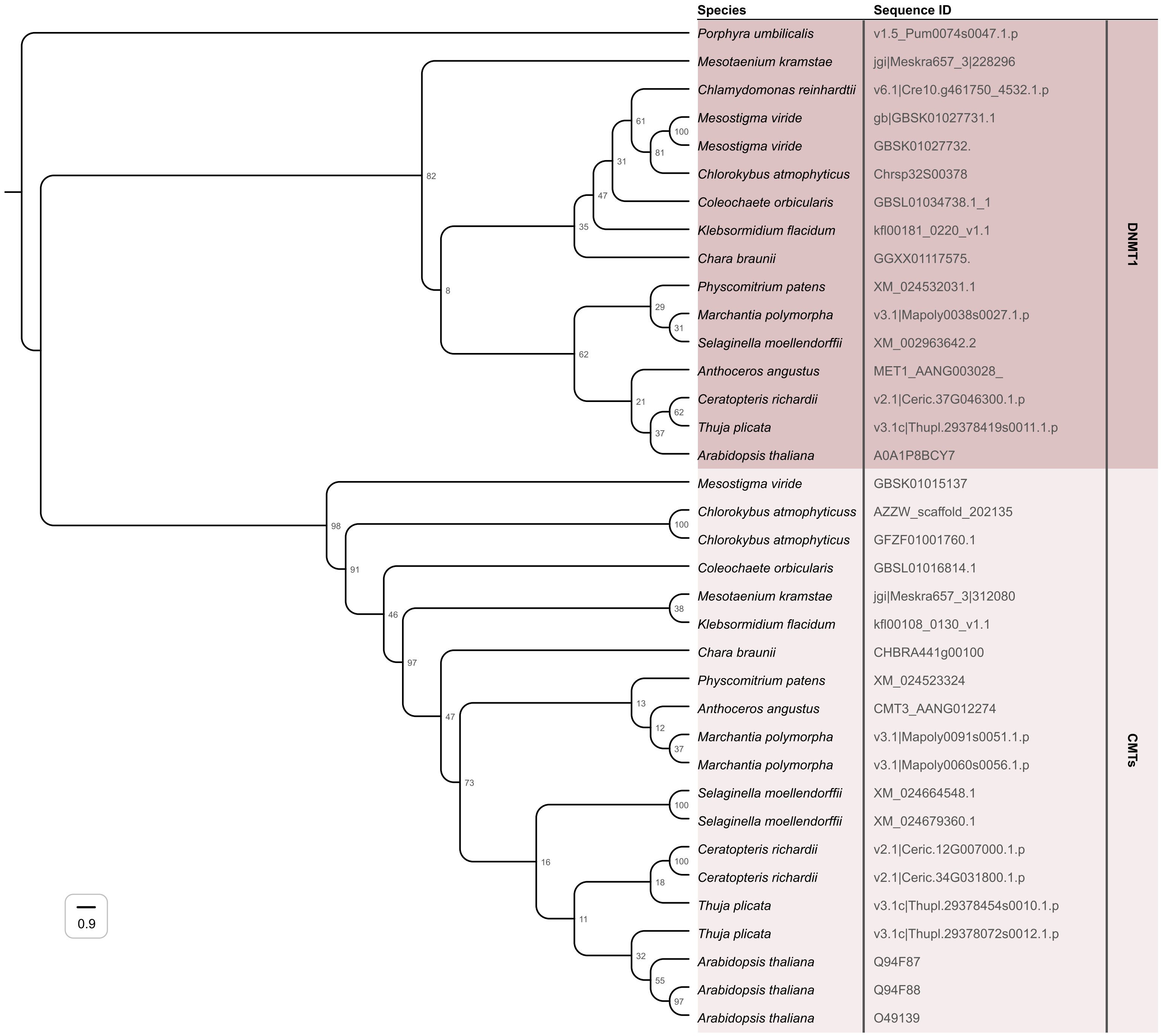
Species
Sequence ID
Porphyra umbilicalis v1.5_Pum0074s0047.1.p
Mesotaenium kramstae jgi|Meskra657_3|228296
Chlamydomonas reinhardtii v6.1|Cre10.g461750_4532.1.p
Mesostigma viride gb|GBSK01027731.1
Mesostigma viride GBSK01027732.
Chlorokybus atmophyticus Chrsp32S00378
Coleochaete orbicularis GBSL01034738.1_1
Klebsormidium flacidum kfl00181_0220_v1.1
Chara braunii GGXX01117575.
Physcomitrium patens XM_024532031.1
Marchantia polymorpha v3.1|Mapoly0038s0027.1.p
Selaginella moellendorffii XM_002963642.2
Anthoceros angustus MET1_AANG003028_
Ceratopteris richardii v2.1|Ceric.37G046300.1.p
Thuja plicata v3.1c|Thupl.29378419s0011.1.p
Arabidopsis thaliana A0A1P8BCY7
Mesostigma viride GBSK01015137
Chlorokybus atmophyticuss AZZW_scaffold_202135
Chlorokybus atmophyticus GFZF01001760.1
Coleochaete orbicularis GBSL01016814.1
Mesotaenium kramstae jgi|Meskra657_3|312080
Klebsormidium flacidum kfl00108_0130_v1.1
Chara braunii CHBRA441g00100
Physcomitrium patens XM_024523324
Anthoceros angustus CMT3_AANG012274
Marchantia polymorpha v3.1|Mapoly0091s0051.1.p
Marchantia polymorpha v3.1|Mapoly0060s0056.1.p
Selaginella moellendorffii XM_024664548.1
Selaginella moellendorffii XM_024679360.1
Ceratopteris richardii v2.1|Ceric.12G007000.1.p
Ceratopteris richardii v2.1|Ceric.34G031800.1.p
Thuja plicata v3.1c|Thupl.29378454s0010.1.p
Thuja plicata v3.1c|Thupl.29378072s0012.1.p
Arabidopsis thaliana Q94F87
Arabidopsis thaliana Q94F88
Arabidopsis thaliana O49139
DNMT1
CMTs
0.9

Fig. S4H

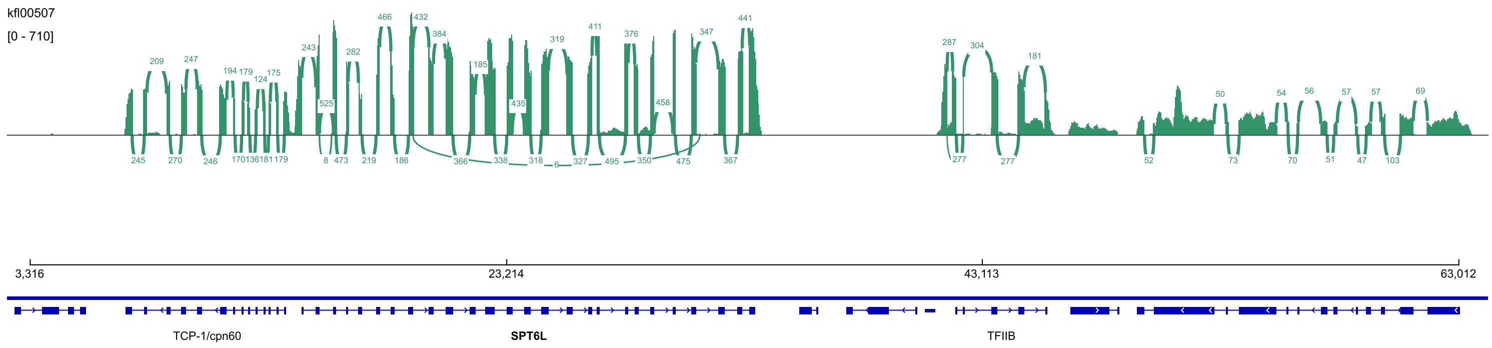

Fig. S4I

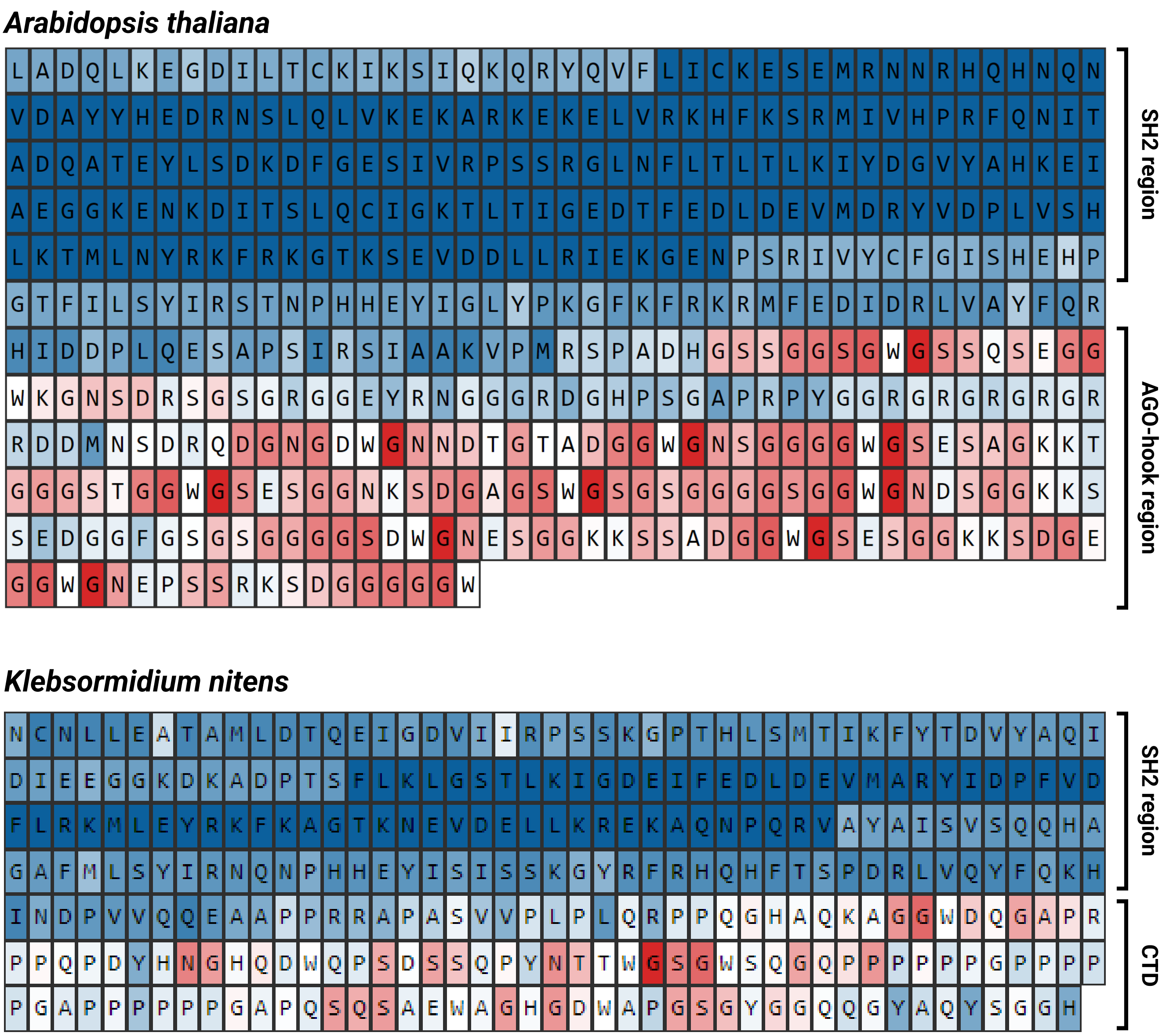

Supplement: Supplementary file 1 [file DataSheet_1.pdf]
